# Phylogenetic Evidence That Two Distinct *Trichuris* Genotypes Infect both Humans and Non-Human Primates

**DOI:** 10.1371/journal.pone.0044187

**Published:** 2012-08-28

**Authors:** Damiana F. Ravasi, Mannus J. O’Riain, Faezah Davids, Nicola Illing

**Affiliations:** 1 Department of Zoology, University of Cape Town, Rondebosch, South Africa; 2 Department of Molecular and Cell Biology, University of Cape Town, Rondebosch, South Africa; Hospital for Sick Children, Canada

## Abstract

Although there has been extensive debate about whether *Trichuris suis* and *Trichuris trichiura* are separate species, only one species of the whipworm *T. trichiura* has been considered to infect humans and non-human primates. In order to investigate potential cross infection of *Trichuris* sp. between baboons and humans in the Cape Peninsula, South Africa, we sequenced the ITS1-5.8S-ITS2 region of adult *Trichuris* sp. worms isolated from five baboons from three different troops, namely the Cape Peninsula troop, Groot Olifantsbos troop and Da Gama Park troop. This region was also sequenced from *T. trichiura* isolated from a human patient from central Africa (Cameroon) for comparison. By combining this dataset with Genbank records for *Trichuris* isolated from other humans, non-human primates and pigs from several different countries in Europe, Asia, and Africa, we confirmed the identification of two distinct *Trichuris* genotypes that infect primates. *Trichuris* sp. isolated from the Peninsula baboons fell into two distinct clades that were found to also infect human patients from Cameroon, Uganda and Jamaica (named the CP-GOB clade) and China, Thailand, the Czech Republic, and Uganda (named the DG clade), respectively. The divergence of these *Trichuris* clades is ancient and precedes the diversification of *T. suis* which clustered closely to the CP-GOB clade. The identification of two distinct *Trichuris* genotypes infecting both humans and non-human primates is important for the ongoing treatment of *Trichuris* which is estimated to infect 600 million people worldwide. Currently baboons in the Cape Peninsula, which visit urban areas, provide a constant risk of infection to local communities. A reduction in spatial overlap between humans and baboons is thus an important measure to reduce both cross-transmission and zoonoses of helminthes in Southern Africa.

## Introduction

Helminths are the most common parasites infecting humans in developing countries and can cause malnutrition, anaemia, growth retardation, and increased susceptibility to other infections [1]. Many pathogens that infect humans and domesticated animals can infect more than one host species [2], [3] and according to Taylor *et al.* [3], 96% of the 287 helminth species found in humans are zoonotic. As urbanization and human population growth forces humans and wild animals into closer and more frequent contact, there are increasing concerns that cross infection of parasites between different primate hosts may lead to the emergence of new diseases in both humans and non-human primates [4]–[6]. This itself is a subject of debate, as although some diseases such as measles and HIV have emerged recently in urbanized human populations, molecular dating suggests that other diseases such as infection by tapeworms, leprosy, and treponematosis have Paleolithic origins [7].

The helminth *Trichuris trichiura* (whipworm) is considered the third most common roundworm to infect humans with an estimated 600 million people infected worldwide [8]. Infection is direct and caused by the ingestion of embryonated eggs from contaminated hands, food, soil or water. After *T. trichiura* eggs have been swallowed, the larvae hatch in the small intestine before travelling to the large intestine where they grow into adult whipworms [9]. The diagnosis of *T. trichiura* is typically done by microscopic visualization of the characteristic lemon shaped eggs in the faeces. The eggs measure 50–55 µm by 22–24 µm, are dark brown in colour and present “plug like” prominences at each pole [10].

Three *Trichuris* species, namely *T. trichiura, T. suis*, and *T. vulpis* are considered zoonotic parasites which are a threat to human health [3]. In the Cape Peninsula (Western Cape Province, South Africa), where informal settlements of humans are characterized by overcrowding and inadequate sanitation, *T. trichiura* is the predominant helminth, with prevalence averaging 51% in children [11]. In parallel, a high prevalence (66%) of *Trichuris* sp. has been recorded in the local population of chacma baboon (*Papio ursinus*) [12]. This geographically isolated and protected baboon population lives in close proximity to urban populations [13], many of which have inadequate sanitation, which raises the risk of zoonotic infection from contaminated water and soil.

Whipworms found in parasitological surveys in non-human primates are typically assumed to belong to the species *T. trichiura*, the whipworm known to infect humans [14], [15]. However, due to the limited external characters of parasites, delimiting species using morphological criteria alone is not accurate [16]. Morphological parameters cannot be used to distinguish between adult females of *T. trichiura* and *T. suis* [17]. Furthermore there is disagreement on whether spicule length in males is longer [17] or shorter [18] in *T. trichiura* compared to *T. suis*. Detailed morphological analyses of *Trichuris* sp. recovered from non-human primates are rare. Ooi *et al.* [14] compared the morphology of *Trichuris* sp. worms collected from macaques (*Macaca fuscata*) and baboons (*Papio papio*) to *T. trichiura* collected from humans, using light and scanning electron microscopy, and concluded that it was not possible to distinguish between these *Trichuris* sp. on morphological grounds. It thus remains uncertain whether *T. trichiura* reported in captive and wild populations of non-human primates are one or more different species.

Molecular techniques are increasingly used as the main tool in the identification of species [19]. Cutillas *et al.* [20] used the internal transcribed spacers (ITS) of the ribosomal DNA to prove the existence of two separate *Trichuris* species in murid and arvicolid rodents. This sequence has also been shown to be a reliable marker to distinguish between *T. suis* isolated from swine or wild boar, *T. vulpis* isolated from dogs [21], and *T. trichiura* isolated from the non-human primates *Colobus guereza kikuyensis* and *Nomascus gabriellae* [17].

In this study the ITS1-5.8S-ITS2 region of ribosomal DNA was sequenced from adult *Trichuris* sp. worms isolated from five baboons from the Cape Peninsula, and from two adult *T. trichiura* isolated from a human patient in Cameroon to determine the genetic relationship between *Trichuris* sp. infecting baboons in the Cape Peninsula and humans. By combining this dataset with Genbank records for *Trichuris* sp. isolated from other humans, non-human primates and pigs from several different countries in Europe, Asia, and Africa, we show that two genetically distinct *Trichuris* genotypes infect humans and non-human primates.

## Methods

### Sample Collection

**Figure 1:**
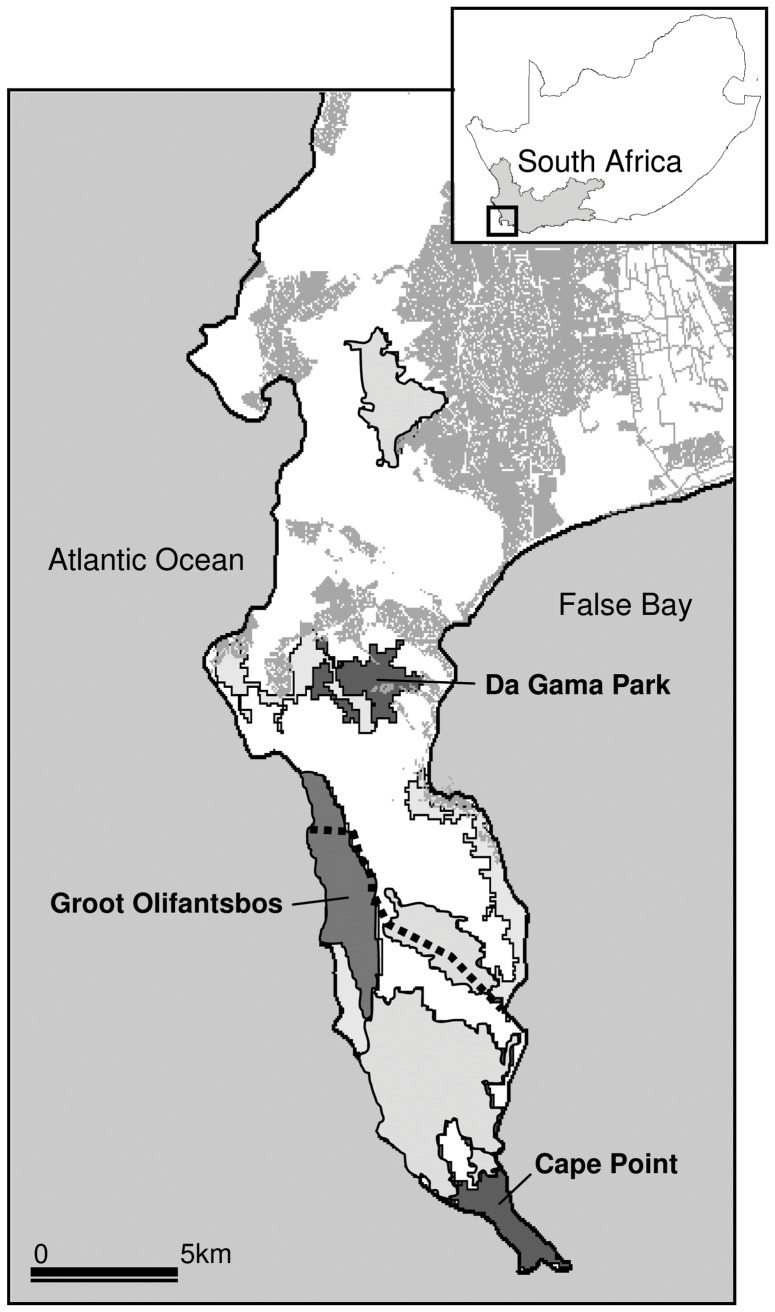
Home ranges of the baboon troops in the Cape Peninsula, South Africa, from which *Trichuris* sp. specimens were collected. Urban areas are shaded in grey and the border of Cape of Good Hope section of the Table Mountain National Park is outlined (dashed line).

*Trichuris* sp. specimens were collected from chacma baboons (*Papio ursinus*) that range in the Cape Peninsula, an area of 470 km^2^ at the south western tip of the African continent, which stretches from the city of Cape Town to the Cape of Good Hope section of the Table Mountain National Park (Fig. 1). Chacma baboons are the only non-human primate species found on the peninsula and are currently protected by legislation. Historically they occurred throughout the peninsula, but agricultural and urban development resulted in a dramatic reduction in suitable natural habitat and a concomitant increase in the frequency and extent of close contact between extant troops and densely populated suburban areas [22]–[24].

We sampled three of the existing 16 Peninsula baboon troops. The Cape Point troop ranges entirely in the Cape of Good Hope section of the Table Mountain National Park (Fig. 1). This troop’s home range overlaps with the Cape Point visitors centre and thus troop members experience frequent, close contact with both local and overseas tourists. The Groot Olifantsbos troop resides in the northernmost section of Cape of Good Hope and their home range overlaps with a residential suburb (Scarborough) that is frequently raided by all troop members (Fig. 1). The Da Gama Park troop is immediately northeast of the Groot Olifantsbos troop and it too overlaps with a residential area bringing troop members into frequent close contact with humans, their waste and a variety of domestic animals [22], [24] (Fig. 1). Data on *Trichuris* sp. prevalence were obtained from 21 and 114 faecal samples collected from the Cape Peninsula and Da Gama Park troops, respectively, between July 2006 and May 2008. Faecal samples were processed using a modified formalin ether sedimentation technique [25] and *Trichuris* sp. eggs were identified and counted. Prevalence was calculated as the percentage of samples with positive *Trichuris* sp. identification.

Opportunistic necropsies were performed whenever dead baboons were brought to the University of Cape Town, South Africa, by the relevant conservation authorities. Adult specimens of the nematode *Trichuris* sp. were collected from the large intestines of three adult females of the Da Gama Park troop (samples DGI, DGII and DGIII), one adult male from the Cape Point troop (sample CP), and one infant female from the Groot Olifantsbos troop (sample GOB). The five worms were stored separately in 70% ethanol and were washed extensively in a saline solution of 0.9% sodium chloride, prior to DNA extraction. Two archived adult specimens of *T. trichiura* were obtained from a previous study [26] collected from a human patient in 2000, from the city of Kumba, Cameroon, following administration of a dose of the antihelminthic drug pyrantel. These specimens were stored at −80°C in a single vial at the University of Nottingham.

### DNA Extraction and Sequencing of the ITS1-5.8S-ITS2 Region

It was not possible to extract DNA from *Trichuris* sp. eggs collected in this study as they had been preserved in formalin. DNA was extracted from whole specimens of *Trichuris* sp. obtained from baboons at the University of Cape Town according to the Tissue Protocol of the QIAamp DNA Mini Kit (Qiagen, Venlo, Netherlands) with the following modification: after overnight incubation at 56°C, two steel beads were added to the lysate solution and subjected to strong shaking for 4 min in a Gyromixer (Fast and Fluid Management, Sassenheim, Netherlands) to completely dissociate the tissue.

DNA from specimens of *T. trichiura* isolated from a human patient was extracted according to a standard protocol [27] at the University of Nottingham to prevent the possibility of any cross-contamination between these DNA samples and DNA samples purified from *Trichuris* sp. isolated at the University of Cape Town. The two worms were homogenized and DNA was sequentially purified, twice with phenol:chloroform:isoamyl alcohol (25∶24:1) and once with chloroform only. The DNA was then precipitated with 3M sodium acetate pH 5.2 and 100% ethanol at −20°C overnight, pelleted and washed with 70% ethanol. After a final centrifugation the DNA was air dried, resuspended in 1× TE buffer and quantified using a NanoDrop® Spectrophotometer (NanoDrop Technologies, USA). DNA was stored at −20°C.

The Polymerase Chain Reaction mix used to amplify the ITS1-5.8S-ITS2 region was prepared with 10 µl of 10× PCR buffer, 2 µl of 10 mM dNTP mixture (0.2 mM each), 6 µl of 25 mM magnesium chloride, 5 µl of forward and reverse primers (0.5 µM each), 1.5 µl of DNA template, 0.5 µl of *Taq* DNA polymerase (2.5 units) and autoclaved distilled water to 100 µl. The conditions applied were: 3 min at 94°C, 35 cycles of 1 min at 94°C, 1 min at 55°C, 1 min at 72°C, with a final extension step of 10 min at 72°C. The primers used in this study, namely forward primer NC5 (5′-GTAGGTGAACCTGCGGAAGGATCATT-3′) and reverse primer NC2 (5′-GGTTAGTTTCTTTTCCTCCGCT-3′) correspond to the conserved ends of the ITS1-5.8S-ITS2 region [28]. The reverse primer NC2 was modified by adding two extra Gs to the 5′ end, to assist with TA cloning and to increase the melting temperature. A negative control was included in each set of PCR reactions.

Purification of amplicons from *Trichuris* sp. samples was done using the Wizard® SV Gel and PCR Clean-Up System (Promega, Madison, USA). One µl of amplicon was cloned into *Escherichia coli* (DH5α) using the pGEM®-T Easy Vectors System (Promega, Madison, USA). Single clones were screened for inserts by colony PCR using flanking primers SP6 (5′-ATTTAGGTGACACTATAGAA-3′) and T7 (5′-TAATACGACTCACTATAGGG-3′). Plasmids were purified using a Qiagen Plasmid Midi Kit (Venlo, Netherlands) and an individual clone from each specimen was sent for sequencing to Macrogen Inc. (Seoul, Korea), using the T7 and SP6 primers. The PCR product from the human *T. trichiura* samples was purified with a QIAquick Gel Extraction Kit (Qiagen, Venlo, Netherlands) and cloned into 5-alpha F’Iq competent *E. coli* (NEB, Ipswich, UK) using thepCR^TM^4-TOPO® vector system (Invitrogen, Paisley, UK). Positive clones were selected on LB agar plates containing 100 µg/ml ampicillin prior to verification by colony PCR using the NC5 and NC2 prime pair. The plasmids from five confirmed positive clones were purified using a Qiagen Plasmid Mini Kit and were sequenced using T3 and T7 primers at the Biopolymer Synthesis and Analysis Unit of the University of Nottingham.

### Sequence and Phylogenetic Analyses

The nucleotide sequences were edited in BioEdit (Ibis Biosciences, Carlsbad, USA). Clones which shared 100% identity were merged. Nucleotide sequences for the ITS1-5.8S-ITS2 regions generated in this study were submitted to GenBank, and their accession numbers are listed in Table 1. MUSCLE software [29], [30] was used to align these sequences to other publicly available ITS1-5.8S-ITS2 or ITS1 or ITS2 sequences (listed in Table 1). Phylogenetic trees based on the MUSCLE alignments were constructed with either the Neighbour-Joining method, Maximum Likelihood, or Maximum Parsimony using the MEGA, version 5 [31] software. The evolutionary distances were computed using the Tajima-Nei method [32]. Bootstrap consensus trees were inferred from 1000 replicates in each instance [33], and were rooted with *Trichuris* isolated from cattle and sheep.

**Table 1:** GenBank Accession Numbers for full length ITS1-5.8S-ITS2 and ITS1 and ITS2 regions used in this study.

GenBank ID	*Trichuris* species	Host species	Country	Region	Living conditions	Reference
AB367794	*Trichuris discolor*	*Capricornis crispus*	Japan	ITS1-5.8S rRNA-ITS2	domestic animal	Unpublished
AB367795	*Trichuris discolor*	Cattle	Japan	ITS1-5.8S rRNA-ITS2	domestic animal	Unpublished
JF680987	*Trichuris ovis*	*Ovis aries*	Ireland	ITS1-5.8S rRNA-ITS2	domestic animal	Unpublished
AM993012	*Trichuris suis*	*Sus scrofa domestica*	China	ITS1-5.8S rRNA-ITS2	domestic animal	[8]
AM993016	*Trichuris suis*	*Sus scrofa domestica*	China	ITS1-5.8S rRNA-ITS2	domestic animal	[8]
FM991956	*Trichuris trichiura*	*Colobus guereza kikuyuensis*	Spain	ITS1-5.8S rRNA-ITS2	zoo	[17]
GQ301555	*Trichuris trichiura*	*Homo sapiens*	Cameroon	ITS1-5.8S rRNA-ITS2		This study
AM992981	*Trichuris trichiura*	*Homo sapiens*	China	ITS1-5.8S rRNA-ITS2		[8]
FM991955	*Trichuris trichiura*	*Nomascus gabriellae*	Spain	ITS1-5.8S rRNA-ITS2	zoo	[17]
GQ301554	*Trichuris* sp.	*Papio ursinus*, CP_GOB	South Africa	ITS1-5.8S rRNA-ITS2	wild ranging, urban	This study
GQ301553	*Trichuris* sp.	*Papio ursinus*, DGI	South Africa	ITS1-5.8S rRNA-ITS2	wild ranging, urban	This study
GQ301552	*Trichuris* sp.	*Papio ursinus*, DGII	South Africa	ITS1-5.8S rRNA-ITS2	wild ranging, urban	This study
GQ301551	*Trichuris* sp.	*Papio ursinus*, DGIII	South Africa	ITS1-5.8S rRNA-ITS2	wild ranging, urban	This study
GQ352554	*Trichuris trichiura*	*Homo sapiens*	Thailand	ITS1		Unpublished
AJ781762	*Trichuris suis*	*Sus scrofa domestica*	Spain	ITS1	domestic animal	[17]
AJ783398	*Trichuris suis*	*Sus scrofa scrofa*	Spain	ITS1	wild animal	[17]
JF690949	*Trichuris* sp.	*Chlorocebus aethiops*	Tanzania	ITS2	wild ranging?	Unpublished
JF690950	*Trichuris* sp.	*Chlorocebus aethiops*	Tanzania	ITS2	wild ranging?	Unpublished
JF690944	*Trichuris* sp.	*Chlorocebus sabaeus*	Czech Republic	ITS2	zoo	Unpublished
JF690940	*Trichuris* sp.	*Homo sapiens*	Czech Republic	ITS2		Unpublished
JF690946	*Trichuris* sp.	*Macaca fascicularis*	Czech Republic	ITS2	zoo	Unpublished
AB586133	*Trichuris* sp.	*Macaca fuscata*	Japan	ITS2	wild ranging	Unpublished
JF690945	*Trichuris* sp.	*Macaca silenus*	Czech Republic	ITS2	zoo	Unpublished
JF690948	*Trichuris* sp.	*Pan troglodytes*	Netherlands	ITS2	zoo	Unpublished
JF690942	*Trichuris* sp.	*Papio anubis*	Czech Republic	ITS2	zoo	Unpublished
JF690941	*Trichuris* sp.	*Papio hamadryas*	Czech Republic	ITS2	zoo	Unpublished
JF690943	*Trichuris* sp.	*Theropithecus gelada*	Czech Republic	ITS2	zoo	Unpublished
JN181814	*Trichuris trichiura*	*Homo sapiens*	Uganda	ITS2		[18]
JN181820	*Trichuris trichiura*	*Homo sapiens*	Uganda	ITS2		[18]
JN181822	*Trichuris trichiura*	*Homo sapiens*	Jamaica	ITS2		[18]
JN181826	*Trichuris trichiura*	*Homo sapiens*	Uganda	ITS2		[18]
JN181827	*Trichuris trichiura*	*Homo sapiens*	Uganda	ITS2		[18]
JN181829	*Trichuris trichiura*	*Homo sapiens*	Uganda	ITS2		[18]
JN181831	*Trichuris trichiura*	*Homo sapiens*	Uganda	ITS2		[18]
JN181834	*Trichuris trichiura*	*Homo sapiens*	Uganda	ITS2		[18]
JN181838	*Trichuris trichiura*	*Homo sapiens*	Uganda	ITS2		[18]
JN181840	*Trichuris trichiura*	*Homo sapiens*	Uganda	ITS2		[18]
JN181842	*Trichuris trichiura*	*Homo sapiens*	Uganda	ITS2		[18]
JN181848	*Trichuris trichiura*	*Homo sapiens*	Uganda	ITS2		[18]
JN181850	*Trichuris trichiura*	*Homo sapiens*	Uganda	ITS2		[18]
JN181852	*Trichuris trichiura*	*Homo sapiens*	Uganda	ITS2		[18]
JN181858	*Trichuris trichiura*	*Homo sapiens*	Uganda	ITS2		[18]
JN181860	*Trichuris trichiura*	*Homo sapiens*	Uganda	ITS2		[18]
JN181797	*Trichuris suis*	*Sus scrofa domestica*	Uganda	ITS2	domestic animal	[18]
JN181770	*Trichuris suis*	*Sus scrofa domestica*	Uganda	ITS2	domestic animal	[18]
JN181785	*Trichuris suis*	*Sus scrofa domestica*	Uganda	ITS2	domestic animal	[18]
JN181791	*Trichuris suis*	*Sus scrofa domestica*	Uganda	ITS2	domestic animal	[18]
JN181804	*Trichuris suis*	*Sus scrofa domestica*	Uganda	ITS2	domestic animal	[18]
AJ249966	*Trichuris suis*	*Sus scrofa domestica*	Spain	ITS2	domestic animal	[17]
JF690951	*Trichuris suis*	*Sus scrofa*	Slovakia	ITS2	domestic animal	Unpublished

A question mark is indicated when information on living conditions was not explicit in Genbank records.

**Figure 2:**
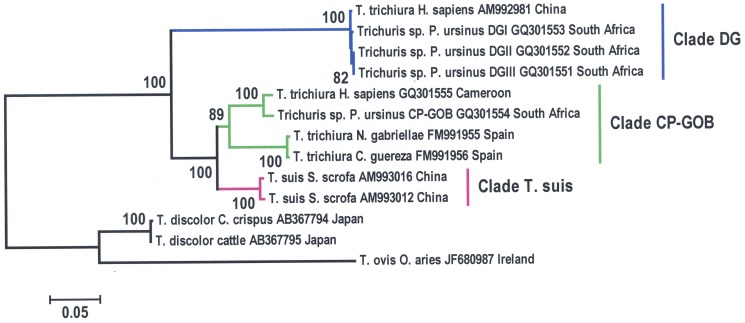
Neighbour-joining phylogenetic tree [**38**] based on a MUSCLE alignment of *ITS1 5.8S-ITS2* sequences from *Trichuris* spp. isolated from human, non-human primates and pigs. Bootstrap values are indicated as percentages on the branches of the consensus tree which was inferred from 1000 replicates [33]. The tree was rooted with *T. ovis and T. discolor*. The evolutionary distances were computed using the Tajima-Nei method [32] and the scale represents the number of base substitutions per site. Branches corresponding to Clade DG are highlighted in blue, while branches corresponding to the Clades CP-GOB and *T. suis* are highlighted in green and pink, respectively.

### Ethics Statement

Research reported here is original and adhered to the American Society of Primatologists Principles for the Ethical Treatment of Non-Human Primates. Data were collected according to protocols approved by the University of Cape Town and South African National Parks, and adhered to the legal requirements of South Africa. A permit was obtained from Cape Research Centre, South African National Parks for both the collection of baboon faeces and the performing of opportunistic necropsies on dead baboons delivered to the University of Cape Town by Conservation Authorities. The permit was valid from January 2007 to January 2012.

The *T. trichiura* worms used in this study were obtained from archived material from a previous study [26]. Verbal informed consent was obtained from the patient, and the guidelines for human experimentations from the Ministry of Health, Cameroon were followed. The study received clearance from the Cameroonian National Ethics Committee. The samples were analyzed anonymously.

## Results

*Trichuris* sp. was one of seven nematode species found in the faeces of the Cape Peninsula baboon troops and had the highest mean (66%) and maximum (98%) prevalence of all nematodes recorded. *Trichuris* eggs from baboons in Da Gama Park measured in average 55.0 (±1.9) um×25.4 (±1.3) um (n = 10) and were found to be smaller than eggs from baboons in Cape Point (64.5 (±2.6) um×30.9 (±2.4) um, (n = 10)). However, a T-test revealed that these differences were not significant.

The ITS1-5.8S-ITS2 region was successfully amplified from genomic DNA from the specimens of *Trichuris* sp. collected from the Peninsula chacma baboons. The sequences (1180 bp) obtained from baboons of the Cape Point and Groot Olifantsbos troops were identical and were thus treated as one sequence, named *Trichuris sp. Papio ursinus CP-GOB* to indicate the troops from which the whipworms were isolated. The ITS1-5.8S-ITS2 sequences (1293 bp) from three specimens of *Trichuris* sp. obtained from three individuals of the Da Gama Park troop shared 98% identity with each other, and were named *Trichuris sp. Papio ursinus DGI, DGII* and *DGIII* respectively. The sequences of the ITS1-5.8S-ITS2 regions isolated from *Trichuris sp*. *DGI-DGIII* were clearly different from the *CP-GOB* sequence and shared only 54% identity.

**Figure 3:**
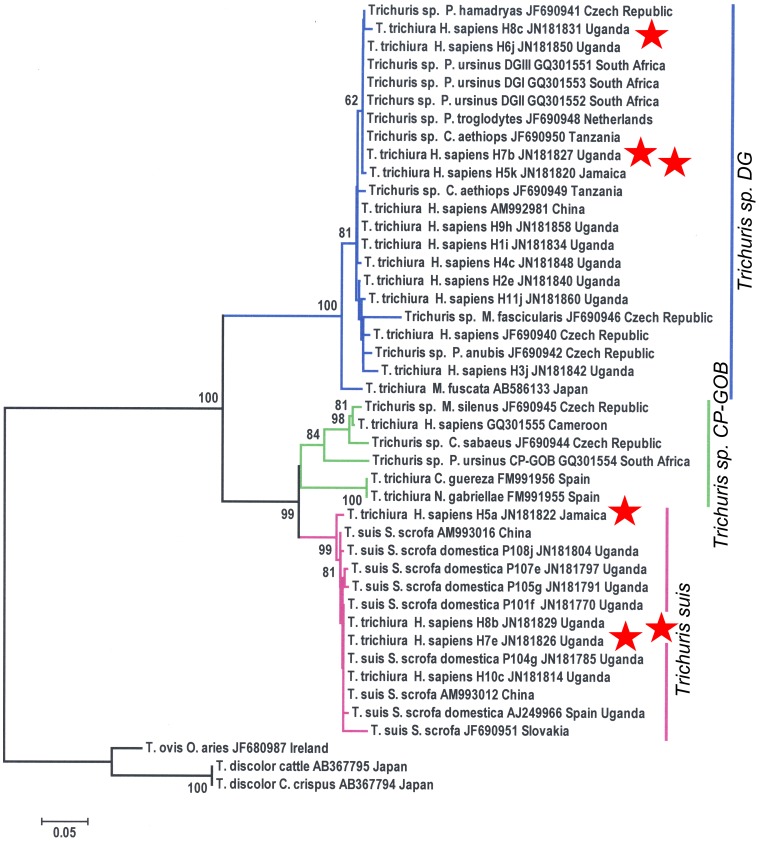
Neighbour-joining phylogenetic tree based on a MUSCLE alignment of *ITS2* sequences from *Trichuris* spp. isolated from human, non-human primates and pigs. Bootstrap values are indicated as percentages on the branches of the consensus tree which was inferred from 1000 replicates [33]. The tree was rooted with *T. ovis and T. discolor*. The evolutionary distances were computed using the Tajima-Nei method [32] and the scale represents the number of base substitutions per site. Branches corresponding to Clade DG are highlighted in blue, while branches corresponding to the Clades CP-GOB and *T. suis* are highlighted in green and pink respectively. Clones H5a and H5k, H7b and H7e, H8b and H8c, derived from the worms H5, H7 and H8 [18] which clustered to different clades respectively, are highlighted with red stars.

Since the sequence for the ITS1-5.8S-ITS2 region for *T. trichiura* isolated from humans was not available at the start of this study, we made use of archived material from a previous study in the Cameroon [26] to investigate the relationship between *Trichuris* sp. isolated from baboons in the Cape Peninsula and *T. trichiura* that infects humans. The *T. trichiura* ITS1-5.8S-ITS2 sequences (1400 bp) of five clones were identical. These sequences shared only 53% identity to the ITS1-5.8S-ITS2 sequence (AM992981) from *T. trichiura* isolated from a human patient in China that was subsequently made available in Genbank.

To our surprise, the *Trichuris sp. Papio ursinus DGI-DGIII* sequences shared 98–99% identity with the ITS1-5.8S-ITS2 sequence from *T. trichiura* isolated from a patient in China. Five variable number of tandem repeats (VNTR) were identified between these sequences, namely DG-VNTR1 ((CAG)_n_ where n ranged from 7, 8 and 10), DG-VNTR2 ((GGC)_n_ where n ranged from 3 to 4), DG-VNTR3 ((CAG)_n_ where n ranged from 7 to 8), DG-VNTR4 ((GAC)_n_ where n ranged from 4 to 5) and DG-VNTR5 ((GGC)_n_ where n ranged from 1 to 2) (Fig. S1). Only six single nucleotide polymorphisms (SNPs) were identified which differed between the *Trichuris sp. DG1-DGIII* and *T. trichiuria (*AM992981*)* sequences (Fig. S1).

However, the sequence of *T. trichiura* isolated from the human patient in Cameroon was similar (91% identity) to the *Trichuris sp. CP-GOB* sequence (Fig. S1). An analysis of the sequence alignment highlights the greater variability between these sequences, with fewer VNTR differences, and many more SNPs and indels (Fig. S1).

Phylogenetic analysis with three different algorithms namely, Neighbour Joining, Maximum Likelihood, and Maximum Parsimony gave the same results (data not shown). *Trichuris sp. DGI-DGIII* clustered into the same clade (Clade DG) as *T. trichiura* isolated from patients in China, while *Trichuris sp. CP-GOB* clustered into the same clade (Clade CP-GOB) as *T. trichiura* isolated from a patient in Cameroon, as well as *Trichuris* sp. isolated from primates *Colobus guereza* and *Nomascus gabriellae* in a Spanish zoo 17 (Fig. 2). The separation of *Trichuris* spp. that infect humans and non-human primates into two genetically distinct clades had robust bootstrap support (Fig. 2). Interestingly, *Trichuris* sp. in Clade CP-GOB are more closely related to *T. suis* (sharing 79% identity), than they are to *Trichuris* sp. in Clade DG (Fig. 2). In addition to having strong bootstrap support, the branch lengths separating *Trichuris sp. CP-GOB* from *T. suis* were longer than the branch lengths separating *Trichuris ovis* from *Trichuris discolour* (Fig. 2). *T. suis* and *Trichuris sp. CP-GOB* are thus likely to be two different species that cluster in clade CP-GOB.

In order to include the largest possible number of sequences available for *Trichuris* species isolated from humans and non-human primates, further phylogenetic analyses were performed using Genbank records for the ITS1 and the ITS2 regions for *T. suis* and *Trichuris* sp. isolated from human and non-human primates (Table 1). The restriction of the analysis to the ITS1 region expanded the dataset to include a *T. trichiura* sequence isolated from a human patient in Thailand, which was grouped into Clade DG, along with the *T. trichiura* from patients in China, whereas *T. suis* from *S. scrofa domestica* and *S. scrofa scrofa* 17 clustered with the other *T. suis* sequences (data now shown).

Phylogenetic analysis of aligned ITS2 *Trichuris* sequences showed that *T. trichiura* isolated from a patient in the Czech Republic, as well as several isolates from patients in Uganda, fell into Clade DG (Fig. 3). *Trichuris* sp. isolated from several species of primates kept in zoos in various countries in Europe and Asia, confirmed that *Trichuris sp.* from both Clade DG and Clade CP-GOB were able to infect non-human primates. All of the *T. suis* ITS2 sequences isolated from pigs around the world, formed a distinct cluster within clade CP-GOB (Fig. 3). The *Trichuris* sp. isolated from patients in Jamaica and Uganda 18 clustered with these *T. suis* sequences, with strong bootstrap support, and short branch lengths, suggesting that recent zoonotic infection may be taking place between pigs and humans living in close proximity in Uganda. Some of the clones in this study 18 had widely divergent sequences with 56–58% identity, even though they were sourced from the same worms, with one sequence clustering to Clade DG and the other sequence clustering to Clade CP-GOB (Fig. 3).

## Discussion

We have isolated and analysed the ITS1-5.8S-ITS2 regions of five *Trichuris* specimens collected from five chacma baboons (*Papio ursinus*) ranging in three different troops in the Cape Peninsula, South Africa. Our results suggest that two distinct *Trichuris* genotypes infect these baboon troops, and that both *Trichuris* genotypes infect humans and non-human primates in Africa, Europe, and Asia. For the purposes of this study, we refer to these two distinct *Trichuris* genotypes as *Trichuris sp. DG* and *Trichuris sp. CP-GOB*, to signify the baboon troops that led to their discovery.

The ITS1-5.8S-ITS2 sequences of the three specimens of *Trichuris* sp. collected from the urban Da Gama Park troop were highly similar to each other (98% identity). The differences between them included three VNTRs and four SNPs. The sequences of the two specimens of *Trichuris* sp., collected from baboons in the neighboring troops of Cape Point and Groot Olifantsbos (Fig. 1) respectively, were identical. The Cape Point and Groot Olifantsbos troops live in the same region of the Cape of Good Hope section of the Table Mountain National Park. Although their home ranges do not directly overlap (Fig. 1), they are indirectly connected by the presence of other troops between them and the regular transfer of adult males between neighboring troops. The ITS1-5.8S-ITS2 sequence of *Trichuris* sp. found in the Da Gama Park troop differed from the *Trichuris* sp. found in the Cape Point and Groot Olifantsbos troops (54% identity).

We compared the ITS1-5.8S-ITS2 regions of our specimens to *T. trichiura* isolated from a patient in Cameroon and to a publicly available sequence of *T. trichiura* from a patient in China. The ITS1-5.8S-ITS2 sequences of *T. trichiura* recovered from humans did not cluster together, and shared only 53% identity. Instead, *T. trichiura* isolated from a patient in China clustered closely to *Trichuris sp. DGI-DGIII* while *T. trichiura* isolated from a patient in Cameroon clustered with *Trichuris sp. CP-GOB*, *T. trichiura N. gabriellae* and *T. trichiura C. guereza* 17.

The levels of genetic divergence were quite different between the clades in our phylogenetic analysis. Whereas the *Trichuris* sequences in Clade DG were all highly similar, with short branch lengths, the *Trichuris* sp. in Clade CP-GOB had much longer branch lengths. *T. suis* was found to cluster within Clade CP-GOB. Our identification of *Trichuris* sp. in clade CP-GOB as a separate genotype from *T. suis* is in agreement with Cutillas *et al.* 17 who concluded that *T. trichiura* isolated from *N. gabriellae* and *C. guereza*, were a distinct species from *T. suis*. Our analysis suggest however that these two genotypes (i.e *T. suis* and *Trichuris* sp. in clade CP-GOB) share a more recent common phylogenetic history than *Trichuris* sp. in clade DG.

An analysis based on only the ITS2 region allowed us to extend our analysis to a larger dataset of 34 publically available sequences, and confirm that two distinct *Trichuris* genotypes infect many species of non-human primates, including hamadryas baboons (*Papio hamadryas*), vervet monkeys (*Chlorocebus aethiops*), and chimpanzees (*Pan troglodytes*). In agreement with Nissen *et al.* 18, some of the ITS2 sequences derived from *T. trichiura* isolated from humans in Uganda, clustered tightly with the *T. suis* ITS2 sequences with little sequence divergence. However, Nissen *et al.* 18 reported that different clones from the same PCR amplification reaction from a single human-derived worm, clustered in two different clades (for example worms H5, H7 and H8). These ITS2 sequences share only 57–58% sequence identity, and are thus unlikely to be “heterozygote” worms as suggested by Nissen *et al.* 18, and may be a consequence of amplification of false PCR products due to cross-contamination of genomic DNA from the *T. suis* samples.

*Trichuris sp. DG* and *Trichuris sp. CP-GOB* may be ancient parasites that have moved between populations of humans and non-human primates for millions of years, having evolved in our hominid ancestors in Africa in the Palaeolithic era 7. It is possible that pigs subsequently became infected with *Trichuris sp. CP-GOB* as a consequence of domestication of animals by humans in the Neolithic era, or alternatively human became infected with *T. suis*, which subsequently infected non-human primates kept under captive conditions in zoos, or whom came into regular contact with humans.

Considering the high prevalence of *Trichuris* sp. in both humans (51%) and baboons (66%) recorded in the Cape Peninsula 11,12, the molecular analysis of *T. trichiura* isolated from human patients in the Cape Peninsula, and baboons both on the Cape Peninsula, and in the wild, far from urban contact, represents the next single most important step in this investigation of patterns of parasite infection. Additionally, the parallel phylogenetic analysis of other genetic loci such as β-tubulin and mitochondrial cytochrome oxidase subunit 1 gene is needed to refine the conclusion that two distinct *Trichuris* genotypes identified in this study are separate species.

Although several authors have concluded that morphological features are uninformative in distinguishing *Trichuris* sp. isolated from humans and non-human primates 14, and between *T. trichiura* and *T. suis* 17,18 these studies grouped all measurements of *Trichuris* isolated from humans and non-human primates and did not take into account the different genotypes of the *Trichuris sp. DG* and *Trichuris sp. CP-GOB*. Our finding of two distinct *Trichuris* genotypes suggests the need for further morphological analyses of adult worms coupled with genetic studies to determine whether the two genotypes reported in this study are distinct species, and whether there are statistically significant morphological features that can be used to distinguish them from each other, and from *T. suis*.

From a human health point of view, baboons could act as a reservoir for primate *Trichuris* sp. in areas with a high degree of contact between baboons and humans, and this remains an important area for future research in the Cape Peninsula. Likely areas for cross transmission of parasites suggested by various authors are: communal water holes 34, human refuse and food wastes, including animal parts 35,36 and soil 37. In the Cape Peninsula, baboons regularly access residential areas and have both direct and indirect (through faeces) contact with gardens and houses, exposing them to a variety of human pathogens 37. From a management perspective, the finding that baboons and humans may both serve as hosts to *Trichuris* sp. and hence could increase infection in one another, provides conservation authorities and town planners alike with a strong argument to restrict the spatial overlap between the human and baboon populations of the Cape Peninsula.

## Supporting Information

Figure S1 MUSCLE alignment of ITS1-5.8S-ITS2 region of ribosomal DNA for *Trichuris* spp. isolated from a range of hosts. Sequence differences between *T. trichiuris H. sapiens* (Cameroon) and *T. trichuris sp P. ursinus CP-GOB*, are highlighted by black rectangles. Single nucleotide polymorphism differences (SNP) between *T. trichuris sp P. ursinus DGI-DGIII* and *T. trichiuris H. sapiens* (China) are indicated by red rectangles. Loci with Variable number of tandem repeats (VNTR) are indicated. The 5.8S region is indicated by a blue rectangle. (PDF)
