# Supplementary material for: Phylogenetic Evidence That Two Distinct Trichuris Genotypes Infect both Humans and Non-Human Primates
Source: PLoS One. 2012 Aug 28;7(8):e44187. doi: 10.1371/journal.pone.0044187 (PMC3429462; doi:10.1371/journal.pone.0044187)
Supplement: Figure S1 — (PDF) [file pone.0044187.s001.pdf]

Figure S1: MUSCLE alignment of *ITS-5.8S-ITS2* for *Trichuris* sp. isolated from a range of hosts. Sequence differences between *T. trichiuris H. sapiens* (Cameroon) and *Trichuris sp P. ursinus CP-GOB*, are highlighted by black rectangles. Single nucleotide polymorphism differences (SNP) between *T. trichiuris H. sapiens* (China) and *Trichrus sp P. ursinus DGI-III* and are indicated by red rectangles. Loci with Variable number of tandem repeats (VNTR) are similarly indicated. The 5.8S region is indicated by a blue rectangle.

```
                                     10        20        30        40        50        60        70        80        90
                            ....|....|....|....|....|....|....|....|....|....|....|....|....|....|....|....|....|....|....|....|

T. trichiura H. sapiens AM9929  ATCAGAAC----------------ACAGCAACAGCAGCACAAAGCGGCCGTAGCTGCCGT--------------------------------------GC
Trichuris sp. P. ursinus DGI G  ATCAGAAC----------------ACAGCAACAGCAGCACAAAGCGGCCGTAGCTGCCGT--------------------------------------GC
Trichuris sp. P. ursinus DGII   ATCAGAAC----------------ACAGCAACAGCAGCACAAAGCGGCCGTAGCTGCCGT--------------------------------------GC
Trichuris sp. P. ursinus DGIII  ATCAGAAC----------------ACAGCAACAGCAGCACAAAGCGGCCGTAGCTGCCGT--------------------------------------GC

T. trichiura H. sapiens GQ3015  ATCAGAACACACACACACACACACACACCAACGGCAACGCAGAGCAGTAG-------CTT--------------------------------------GT
Trichuris sp. P. ursinus CP-GO  ATCAGAAC----------------ACACCAACGGCAACGCAGAGCAGTAG-------CTT--------------------------------------GT
                                 CP-GOB VNTR-1

T. trichiura N. gabriellae FM9  ATCAGAGC----------------ACATTAACGGCAACGCAGGGCAGTAG-------CGT--------------------------------------GC
T. trichiura C. guereza FM9919  ATCAGAGC----------------ACATTAACGGCAACGCAGGGCAGTAG-------CGT--------------------------------------GC

T. suis S. scrofa AM993016 Chi  ATCAGAAC----------------ACACCAACGGCAACGCAGAGCAGTAG-------CGT--------------------------------------GT
T. suis S. scrofa AM993012 Chi  ATCAGAAC----------------ACACCAACGGCAACGCAGAGCAGTAG-------CGT--------------------------------------GT

T. ovis O. aries JF680987 Irel  ATCAGAACTCCAACAACA------ACAACAACCGTTACGAGAAGTTGTTG-------CTTCAAAAGGCTGCTTGAGATTTGGACTGCTGCTGACAGCGGC
T. discolor cattle AB367795 Ja  ATCAGAACTCGAACA---------ACAACAACCGTTACGAGAAGTTGTTG-------CTTCTAAGGGCCGTTCGAGATTTGGACTGCTGCTGGCAGCGGC
T. discolor C. crispus AB36779  ATCAGAACTCGAACA---------ACAACAACCGTTACGAGACGTTGTTG-------CTTCTAAGGGCCGTTCGAGATTTGGACTGCTGCTGGCAGCGGC
```

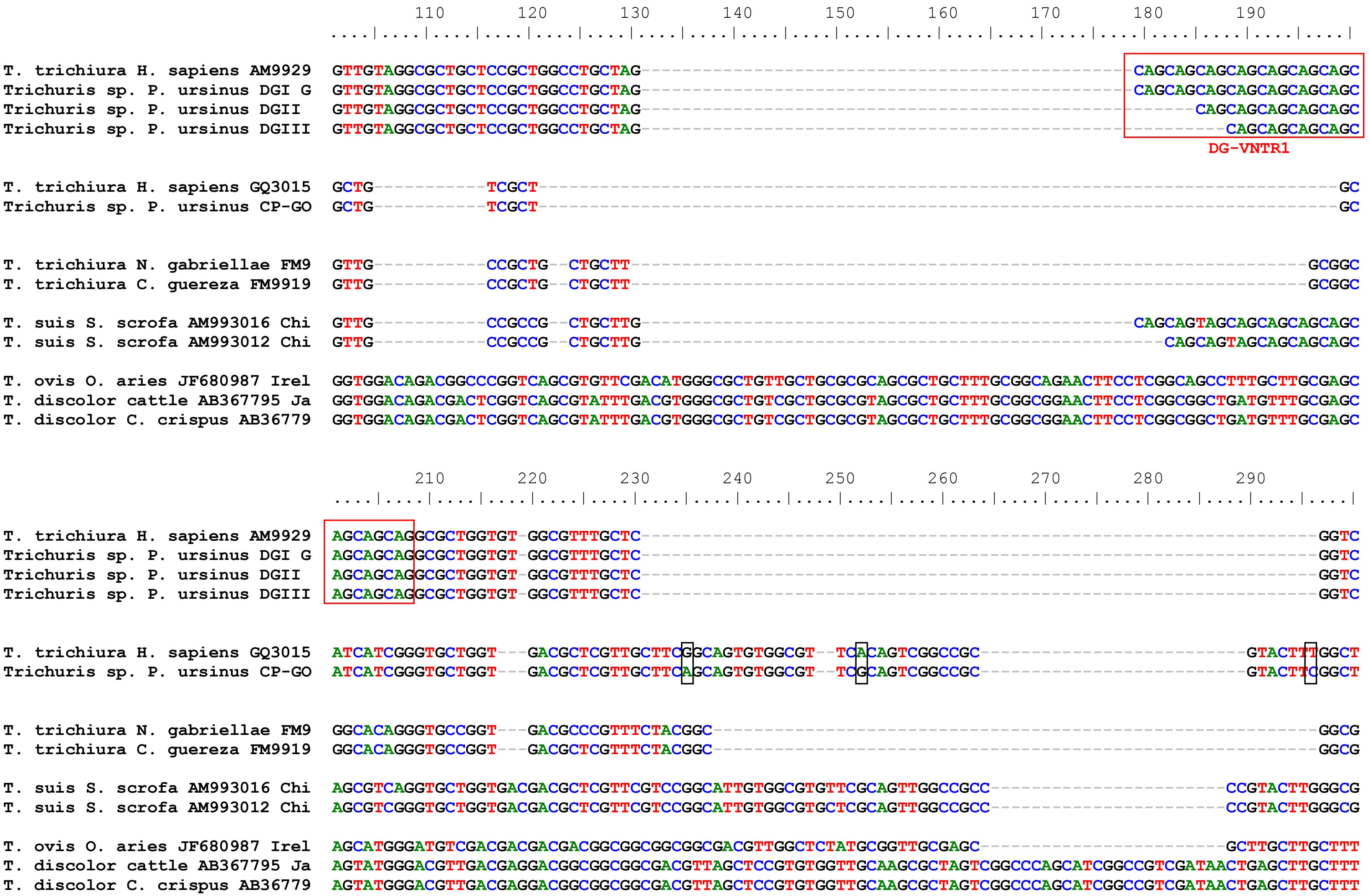
110 120 130 140 150 160 170 180 190
T. trichiura H. sapiens AM9929 GTTGTAGGCGCTGCTCCGCTGGCCTGCTAG --- CAGCAGCAGCAGCAGCAGCAGC
Trichuris sp. P. ursinus DGI G GTTGTAGGCGCTGCTCCGCTGGCCTGCTAG --- CAGCAGCAGCAGCAGCAGCAGC
Trichuris sp. P. ursinus DGII GTTGTAGGCGCTGCTCCGCTGGCCTGCTAG --- CAGCAGCAGCAGCAGC
Trichuris sp. P. ursinus DGIII GTTGTAGGCGCTGCTCCGCTGGCCTGCTAG --- CAGCAGCAGCAGC
DG-VNTR1
T. trichiura H. sapiens GQ3015 GCTG --- TCGCT --- GC
Trichuris sp. P. ursinus CP-GO GCTG --- TCGCT --- GC
T. trichiura N. gabriellae FM9 GTTG --- CCGCTG --- CTGCTT --- GCGGC
T. trichiura C. guereza FM9919 GTTG --- CCGCTG --- CTGCTT --- GCGGC
T. suis S. scrofa AM993016 Chi GTTG --- CCGCCG --- CTGCTTG --- CAGCAGTAGCAGCAGCAGCAGC
T. suis S. scrofa AM993012 Chi GTTG --- CCGCCG --- CTGCTTG --- CAGCAGTAGCAGCAGCAGC
T. ovis O. aries JF680987 Irel GGTGGACAGACGGCCCGGTCAGCGTGTTCGACATGGGCGCTGTTGCTGCGCGCAGCGCTGCTTTGCGGCAGAACTTCCTCGGCAGCCTTTGCTTGCGAGC
T. discolor cattle AB367795 Ja GGTGGACAGACGACTCGGTCAGCGTATTTGACGTGGGCGCTGTCGCTGCGCGTAGCGCTGCTTTGCGGCGGAACTTCCTCGGCGGCTGATGTTTGCGAGC
T. discolor C. crispus AB36779 GGTGGACAGACGACTCGGTCAGCGTATTTGACGTGGGCGCTGTCGCTGCGCGTAGCGCTGCTTTGCGGCGGAACTTCCTCGGCGGCTGATGTTTGCGAGC
210 220 230 240 250 260 270 280 290
T. trichiura H. sapiens AM9929 AGCAGCAGGCGCTGGTGT GGCGTTTGCTC --- GGTC
Trichuris sp. P. ursinus DGI G AGCAGCAGGCGCTGGTGT GGCGTTTGCTC --- GGTC
Trichuris sp. P. ursinus DGII AGCAGCAGGCGCTGGTGT GGCGTTTGCTC --- GGTC
Trichuris sp. P. ursinus DGIII AGCAGCAGGCGCTGGTGT GGCGTTTGCTC --- GGTC
T. trichiura H. sapiens GQ3015 ATCATCGGGTGCTGGT --- GACGCTCGTTGCTTCGGCAGTGTGGCGT --- TCACAGTCGGCCGC --- GTACTTTGGCT
Trichuris sp. P. ursinus CP-GO ATCATCGGGTGCTGGT --- GACGCTCGTTGCTTCAGCAGTGTGGCGT --- TCGCAGTCGGCCGC --- GTACTTCGGCT
T. trichiura N. gabriellae FM9 GGCACAGGGTGCCGGT --- GACGCCCGTTTCTACGGC --- GGCG
T. trichiura C. guereza FM9919 GGCACAGGGTGCCGGT --- GACGCTCGTTTCTACGGC --- GGCG
T. suis S. scrofa AM993016 Chi AGCGTCAGGTGCTGGTGACGACGCTCGTTCGTCCGGCATTGTGGCGTGTTCGCAGTTGGCCGCC --- CCGTACTTGGGCG
T. suis S. scrofa AM993012 Chi AGCGTCGGGTGCTGGTGACGACGCTCGTTCGTCCGGCATTGTGGCGTGCTCGCAGTTGGCCGCC --- CCGTACTTGGGCG
T. ovis O. aries JF680987 Irel AGCATGGGATGTCGACGACGACGACGGCGGCGGCGGCGACGTTGGCTCTATGCGGTTGCGAGC --- GCTTGCTTGCTTT
T. discolor cattle AB367795 Ja AGTATGGGACGTTGACGAGGACGGCGGCGGCGACGTTAGCTCCGTGTGGTTGCAAGCGCTAGTCGGCCCAGCATCGGCCGTCGATAACTGAGCTTGCTTT
T. discolor C. crispus AB36779 AGTATGGGACGTTGACGAGGACGGCGGCGGCGACGTTAGCTCCGTGTGGTTGCAAGCGCTAGTCGGCCCAGCATCGGCCGTCGATAACTGAGCTTGCTTT

```
                                    310       320       330       340       350       360       370       380       390
                              ....|....|....|....|....|....|....|....|....|....|....|....|....|....|....|....|....|....|....|....|

T. trichiura H. sapiens AM9929  CGAT-------------------------GCGAGCAACGTCCGCCG-------GCTT------ACAGTTCCAGGTCTGAAGAGCGCAGGCGGCGGC----
Trichuris sp. P. ursinus DGI G  CGAT-------------------------GCGAGCAACGTCCGCCG-------GCCT------ACAGTTCCAGGTCTGAAGAGCGCAGGCGGCGGCGGC-
Trichuris sp. P. ursinus DGII   CGAT-------------------------GCGAGCAACGTCCGCCG-------GCTT------ACAGTTCCAGGTCTGAAGAGCGCAGGCGGCGGC----
Trichuris sp. P. ursinus DGIII  CGAT-------------------------GCGAGCAACGTCCGCCG-------GCTT------ACAGTTCCAGGTCTGAAGAGCGCAGGCGGCGGC----
                                                                                       SNP1                                   DG-VNTR2

T. trichiura H. sapiens GQ3015  CGACATGAGCGCGCGTTGCCGCTGCCGAGGAGAACGGCGTGCACGGTTGCAGCACCA------GCACTTCCAGATCCGAAGAGCA--GGCCGCGCTGGCT
Trichuris sp. P. ursinus CP-GO  CGACACGAGCGCGCGTTGCCGCTGCTGCCGAGAACAGCGTGCACCGTT---GCACCA------GCACTTCCAGATCCGAAGAGCA--GGCCGCGCTTGCT

T. trichiura N. gabriellae FM9  CGACGTGAGCGCGCGTTGCCACTGCC---GAGAGCAGCGTCCACCGTT---GCACCA------GCAGTTCCAGATCCGAAGAGCA--GGTCGCGCTGACC
T. trichiura C. guereza FM9919  CGACGTGAGCGCGCGTTGCCACTGCC---GAGAGCAGCGTCCACCGTT---GCACCA------GCAGTTCCAGATCCGAAGAGCA--GGTCGCGCTGACC

T. suis S. scrofa AM993016 Chi  CGACGTGAGCGCGCGTTGCCACTGCC---GTGAGCAGCGTCCACCGTT---GCACCA------CCACTTCCAGATCCGAAGCGCA--GGCCGCGCTGGCT
T. suis S. scrofa AM993012 Chi  CGACGTGAGCGCGCGTTGCCACTGCC---GTGAGCAGCGTCCACCGTT---GCACCA------CCACTTCCAGATCCGAAGCGCA--GGCCGCGCTGGCT

T. ovis O. aries JF680987 Irel  CTAC-----CGTGAGCTTTCACCACCGCAGG-----ACGTTCGTCGTTCTTCTACCATGCTGCTTACTTCCAGATCTTAAGGC----GATCGCTCCCGCT
T. discolor cattle AB367795 Ja  CTAC-----CGTGAGCTTTCACCACCGCAGC-----ATGTTCGCCCTTGTTCTACCATGCTGCTTACTTCCAGATCTTAAGGC----GAACGCTCCCGTT
T. discolor C. crispus AB36779  CTAC-----CGTGAGCTTTCACCACCGCAGC-----ATGTTCGCCCTTGTTCTACCATGCTGCTTACTTCCAGATCTTAAGGC----GAACGCTCCCGTT

                                     410       420       430       440       450       460       470       480       490
                              ....|....|....|....|....|....|....|....|....|....|....|....|....|....|....|....|....|....|....|....|

T. trichiura H. sapiens AM9929  ----TCGTTC-CGCCCGGCG-GGCGAGTCGTCGATCGTCGCTGTCACCGCC---------------------------TCGATCAGGCAGCAGCAATTTT
Trichuris sp. P. ursinus DGI G  ----TCGTTC-CGCCCGGCG-GGCGAGTCGTCGATCGTCGCCGTCACCGGC---------------------------TCGATCAGGCAGCAGCGGTTTT
Trichuris sp. P. ursinus DGII   ----TCGTTC-CGCCCGGCG-GGCGAGTCGTCCATCGTCGCCGTCACCGGC---------------------------TCGATCAGGCAGCAGCAATTTT
Trichuris sp. P. ursinus DGIII  ----TCGTTC-CGCCCGGCG-GGCGAGTCGTCGATCGTCGCCGTCACCGGC---------------------------TCGATCAGGCAGCAGCAATTTT
                                                                        SNP2                                                  SNP3-4

T. trichiura H. sapiens GQ3015  ACGGTCGTCC-CGCGCTGCTCGACGACTACGTGGCTACCGCCAGTACGACCGGCTGCAGACGGACGACTGCTGCTGCGTAGCTCAGTCAGCTAGCACCAG
Trichuris sp. P. ursinus CP-GO  ACGGTCGTCC-CGCGCTGCTCGACGACTACGCGGCTACCGCCAGT-CGACCGGCTGCAGACGAACGA---CTGCGTAGTAGCTCAGGCAGCTAGCACCAG

T. trichiura N. gabriellae FM9  ACGGTCGACC-TGCGCCGCT---CGACACGGTGACCACCAGCAGTACGGCCGGCTGCAGACGGACGA---CTGC---GCGGCTGAGTCAGCCAGCATCGT
T. trichiura C. guereza FM9919  ACGGTCGACC-TGCGCCGCT---CGACACGGTGACCACCAGCAGTACGGCCGGCTGCAGACGGACGA---CTGC---GCGGCTGAGTCAGCCAGCATCGT

T. suis S. scrofa AM993016 Chi  ACGGTCGTCC-TGCGCCGCT---TGACTCGGTGATCACCACCAGTACGACCGGCTGCAGACGGACGA---CTGC---GTGGCTGAGTCAGTCGGCATCGT
T. suis S. scrofa AM993012 Chi  ACGGTCCTCC-TGCGCCGCCGCTTGACTCGGTGATCACCACCAGTACGACCGGCTGCAGACGGACGA---CTGC---GTGGCTGAGTCAGTCGGCATCGT

T. ovis O. aries JF680987 Irel  ACCACCGTGAAAGCTCGGAGCAAC----------------------CGTTCAGCCACAGCTG--------CGGTCGTGTAGCTCAG-CTGCTGGTGGAAA
T. discolor cattle AB367795 Ja  ATCACCAGGCAAGCTCGGCAAGCATGGTACGCGATCAAC-------CGTTCAGCCTCAGCTG--------CGGTCGCGTAGCTCAG-CTCCTGGTGGCAA
T. discolor C. crispus AB36779  ATCACCAGGCAAGCTCGGCAAGCATGGTACGCGATCAAC-------CGTTCAGCCTCAGCTG--------CGGTCGCGTAGCTCAG-CTCCTGGTGGCAA
```

```
                                          510       520       530       540       550       560       570       580       590
                                 ....|....|....|....|....|....|....|....|....|....|....|....|....|....|....|....|....|....|....|....|

T. trichiura H. sapiens AM9929   CA-------------------CTTGCTGCTCGCCGCCTGCGCGCAGT---CACCGGTACCTGTTCCGTTGTGGCCTCCGTGGCTGCGGCAGTTTGGATCT
Trichuris sp. P. ursinus DGI G   CA-------------------CTTGCTGCTCGCCGCCTGCGCGCAGT---CACCGGTACCTGTTCCGTTGTGGCCTCCGTGGCTGCGGCAGTTTGGATCT
Trichuris sp. P. ursinus DGII    CA-------------------CTTGCTGCTCGCCGCCTGCGCGCAGT---CACCGGTACCTGTTCCGTTGTGGCCTCCGTGGCTGCGGCAGTTTGGATCT
Trichuris sp. P. ursinus DGIII   CA-------------------CTTGCTGCTCGCCGCCTGCGCGCAGT---CACCGGTACCTGTTCCGTTGTGGCCTCCGTGGCTGCGGCAGTTTGGATCT

T. trichiura H. sapiens GQ3015   CACCGGCAGCCGCTAGACGTCCGTGCTCGCGCGCGCCTGCCAACAACGATGACCGGTACCTGTTCCGTCTGGGCCTCAGTAGCTGCGGCAGTGTGGATCT
Trichuris sp. P. ursinus CP-GO   CA------GCCGCTAGGCGTCCGTGCT--CGCGCGCCTGCCAACAACGATGACCGGTACCTGTTCCGTCTGGGCCTCAGTAGCTGCGGCAGTGTGGATCT

T. trichiura N. gabriellae FM9   CG-----AGCGGCCGGACGTCCGTGTT--CCCGCGCCTGCCAACAACGATGACCGGTACCTGCTCCGTCTGGGCCTCAGTAGCTGCGGCAGTGTGGATCT
T. trichiura C. guereza FM9919   CG-----AGCGGCCGGACGTCCGTGTT--CCCGCGCCTGCCAACAACGATGACCGGTACCTGCTCCGTCTGGGCCTCAGTAGCTGCGGCAGTGTGGATCT

T. suis S. scrofa AM993016 Chi   CGCGG----------GACGTCCGTGCT--TGCTCGCCTGCCAACAAC---GACCGGTACCTGTTCCGTCTGGGCCTCAGTGGCTGCGGCAGTGTGGATCT
T. suis S. scrofa AM993012 Chi   CGCGG----------GACGTCCGTGCT--TGCTCGCCTGCCAACAAC---GACCGGTACCTGTTCCGTCTGGGCCTCAGTGGCTGCGGCAGTGTGGATCT

T. ovis O. aries JF680987 Irel   TGCGGTTAG------------CGCGTTTTATTCGCCT---------TGATAGACGGTACCTGTTCCGTTTGGGCTTCACTGGCAGCGGCAGTGTAGATCT
T. discolor cattle AB367795 Ja   TGCGGTTAG------------CGCGTT--TATTCGCCT---------TGATAGACGGTACCTGTTCCGTTTGGGCTTCACTGGCAGCGGCAGTGTAGATCT
T. discolor C. crispus AB36779   TGCGGTTAG------------CGCGTT--TATTCGCCT---------TGATAGACGGTACCTGTTCCGTTTGGGCTTCACTGGCAGCGGCAGTGTAGATCT

                                          610       620       630       640       650       660       670       680       690
                                 ....|....|....|....|....|....|....|....|....|....|....|....|....|....|....|....|....|....|....|

T. trichiura H. sapiens AM9929   GGCTGTCGTTAGCGCCGCCGGTTGCAGTCGACCGTGCTGCCCCGC------CA--GTAGTTGCAC-----------TGGCGCTGAAGAAGCAGCGGCTAG
Trichuris sp. P. ursinus DGI G   GGCTGTCGTTAGCGCCGCCGGTTGCAGTCGACCGTGCTGCCCCGC------CA--GTAGTTGCAC-----------TGGCGCTGAAGAAGCAGCGGCTAG
Trichuris sp. P. ursinus DGII    GGCTGTCGTTAGCGCCGCCGGTTGCAGTCGACCGTGCTGCCCCGC------CA--GTAGTTGCAC-----------TGGCGCTGAAGAAGCAGCGGCTAG
Trichuris sp. P. ursinus DGIII   GGCTGTCGTTAGCGCCGCCGGTTGCAGTCGACCGTGCTGCCCCGC------CA--GTAGTTGCAC-----------TGGCGCTGAAGAAGCAGCGGCTAG

T. trichiura H. sapiens GQ3015   GGCTGCCGTCAGCGCCGCCGGTTGCAACCGACCGTGCTGCTGCGG-GCTGTCATCA-CGTCGCCGACCGCCCCCGGTGGCGTTGATGATGC---GGCATG
Trichuris sp. P. ursinus CP-GO   GGCTGCCGTCAGCGCCGCCGGTTGCAACCGACCTTGCTGCTGCGG-GCTGTCATCA-CGTCGCCGACCGCCCCCGGTGGCGTTGATGATGC---GGCA-G

T. trichiura N. gabriellae FM9   GGCTGCCGTCAGCGCCGCCGGTTGCAGCCGACCGTGCCGTCGCGA-GCTGTCATCA-CGTCGCCGACCGCTCCCGGTGGCGTTGATGAAGCGGCGGCACG
T. trichiura C. guereza FM9919   GGCTGCCGTCAGCGCCGCCGGTTGCAGCCGACCGTGCCGTCGCGA-GCTGTCATCA-CGTCGCCGACCGCTCCCGGTGGCGTTGATGAAGCGGCGGCACG

T. suis S. scrofa AM993016 Chi   GGCTGCCGTTAGCGCCGCCGGTTGCAGCCGACCGTGCTGCTGCGG-GTTTTCATCA-CGTCGCCGAGC-CCCCCGGTGGCGTCGATGAA---GCGGCATG
T. suis S. scrofa AM993012 Chi   GGCTGCCGTTAGCGCCGCCGGTTGCAGCCGACCGTGCTGCTGCGG-GTTTTCATCA-CGTCGCCGAGC-CCCCCGGTGGCGTCGATGAA---GCGGCATG

T. ovis O. aries JF680987 Irel   GGCTGCCGCTTGCGCCGCCGGTTGCAGACGACAACGTTGCTTCAGTTCCGTGATCAGCTTCGATGCATCTCCGGAGCGGAGACGCTACGACGCGGACTGG
T. discolor cattle AB367795 Ja   GGCTGTCGCTTGCGCCGCCGGTTGCAGACGACTGCGCTGCTTCAGTTCCGTGCTCAGCTTCGATGTATCTTCGGAGCGGAGACGCTACGATACGGACTGG
T. discolor C. crispus AB36779   GGCTGTCGCTTGCGCCGCCGGTTGCAGACGACTGCGCTGCTTCAGTTCCGTGCTCAGCTTCGATGTATCTTCGGAGCGGAGACGCTACGATACGGACTGG
```

```
                                          710       720       730       740       750       760       770       780       790
                                 ....|....|....|....|....|....|....|....|....|....|....|....|....|....|....|....|....|....|....|....|

T. trichiura H. sapiens AM9929   C---G---------TCGACGAGGTTCAAAGAACAGCCGTT-----CGACCTCGAGC--ATCGAGG-------ACGGCTCGTCGCCGGTTGGAAAAAGAAA
Trichuris sp. P. ursinus DGI G   C---G---------TCGACGAGGTTCAAAGAACAGCCGTT-----CGACCTCGAGC--ATCGAGG-------ACGGCTCGTCGCCGGTTGGAAAAAGAAA
Trichuris sp. P. ursinus DGII    C---G---------TCGACGAGGTTCAAAGAACAGCCGTT-----CGACCTCGAGC--ATCGAGG-------ACGGCTCGTCGCCGGTTGGAAAAAGAAA
Trichuris sp. P. ursinus DGIII   C---G---------TCGACGAGGTTCAAAGAACAGCCGTT-----CGACCTCGAGC--ATCGAGG-------ACGGCTCGTCGCCGGTTGGAAAAAGAAA

T. trichiura H. sapiens GQ3015   CTATG---------TCGGTGAGGTTTAAAGAACGGCCGTT-----CG-CCTCCGTGAGCGAATCGGGGAAGGACGGCTCGTCGCCGGTTGG---------
Trichuris sp. P. ursinus CP-GO   CTATG---------TCGGTGAGGTTTAAAGAACGGCCGTT-----CG-CCTCCATGAGCGAATCGGGGAAGGACGGCTCGTCGCCGGTTGG---------

T. trichiura N. gabriellae FM9   ACACG---------TCGGTGAGGTGTAAGGAACGGCCGTT-----CG-CCACCTTGAGCGCACAAGAAAGCAACGGCTCGTCGCCGGTTGG---------
T. trichiura C. guereza FM9919   ACACG---------TCGGTGAGGTGTAAGGAACGGCCGTT-----CG-CCACCTTGAGCGCACAAGAAAGCAACGGCTCGTCGCCGGTTGG---------

T. suis S. scrofa AM993016 Chi   CTATG---------TCGGTGAGGTTTAAAGAACGGCCGTT-----CGAACTCGAGCGCATCG-AGGAACGCGACGGCTCGTCGCCGGTTGG---------
T. suis S. scrofa AM993012 Chi   CTATG---------TCGGTGAGGTTTAAAGAACGGCCGTT-----CGAACTCGAGCGCATCG-AGGAACGCGACGGCTCGTCGCCGGTTGG---------

T. ovis O. aries JF680987 Irel   CTGCGCGTATACGTTCGTCGAGGTTCAAAGAACGACTGTCGTGGGCGCCACGCTTGGCGCATCCA----TTGACGGATCGTCGCCGTTTGG---------
T. discolor cattle AB367795 Ja   CTGCGCGTATACGTTCGTCGAGGTTCAAAGAACGACTGTCGAGAGTGCGAGGCTTGGCGCACCCT----TCGACGGTTCGTCGCCGTCTGG---------
T. discolor C. crispus AB36779   CTGCGCGTATACGTTCGTCGAGGTTCAAAGAACGACTGTCGAGAGTGCGAGGCTTGGCGCACCCT----TCGACGGTTCGTCGCCGTCTGG---------


                                          810       820       830       840       850       860       870       880       890
                                 ....|....|....|....|....|....|....|....|....|....|....|....|....|....|....|....|....|....|....|

T. trichiura H. sapiens AM9929   AAGCAGCTTC---------ACCAGTGCACA-AGG----CGTCGTCCTACTACGCACGCCGATGTGCCTTCGACACGCAGACACTGCTGTCGGCGCAATGA
Trichuris sp. P. ursinus DGI G   AAGCAGCTTC---------ACCAGTGCACA-AGG----CGTCGTCCTACTACGCACGCCGATGTGCCTTCGACACGCAGACACTGCTGTCGGCGCAATGA
Trichuris sp. P. ursinus DGII    AAGCAGCTTC---------ACCAGTGCACA-AGG----CGTCGTCCTACTACGCACGCCGATGTGCCTTCGACACGCAGACACTGCTGTCGGCGCAATGA
Trichuris sp. P. ursinus DGIII   AAGCAGCTTC---------ACCAGTGCACA-AGG----CGTCGTCCTACTACGCACGCCGATGTGCCTTTGACACGCAGACACTGCTGTCGGCGCAATGA
                                                                                                      SNP5

T. trichiura H. sapiens GQ3015   ---ATCTTCC---TAAATGACCAATGCAGATAAG----CTGCGCTT---------CGCTGAAG-----------CGTAGGAACGAATTGTTGAACG----
Trichuris sp. P. ursinus CP-GO   ---ATCTTCG---TTCATGACCAATGCAGATAAG----CTGCGCTT---------CGCTGAAGCGA--------CGCAGGAACGAATTGTTGAACT----

T. trichiura N. gabriellae FM9   ---ATCTCCG---TTTACGACCAATGCAGATAAG----CTGCGCTT---------CGCTGAAG-----------CGCAGGAACGAACTGTTGAGCGATGA
T. trichiura C. guereza FM9919   ---ATCTCCG---TTTACGACCAATGCAGATAAG----CTGCGCTT---------CGCTGAAG-----------CGCAGGAACGAACTGTTGAGCGATGA

T. suis S. scrofa AM993016 Chi   ---ATCTTCGTTATTAACGACCAATGCAGATAAG----CTGCGCTT---------CGTCGAAG-----------TGCAGGAACTC---TTGAAAC-----
T. suis S. scrofa AM993012 Chi   ---ATCTTCGTTATTAACGACCAATGCAGATAAG----CTGCGCTT---------CGTCGAAG-----------TGCAGGAACTC---TTGAAAC-----

T. ovis O. aries JF680987 Irel   -------------------ACCAGTACGCTTCGGTCCACTTCACACTA-------CGCAACAACAGCGTTGCTCTGTATGAATTCGAATTGGGAAAGCAA
T. discolor cattle AB367795 Ja   -------------------ACCAGTACATTTCGGTCCACTTCACTCTA-------CGCAACAACAGCGTTGCTCTGTACGAATTCGAACTGGGAAAGCAA
T. discolor C. crispus AB36779   -------------------ACCAGTACATTTCGGTCCACTTCACTCTA-------CGCAACAACAGCGTTGCTCTGTACGAATTCGAACTGGGAAAGCAA
```

```
                                              910       920       930       940       950       960       970       980       990
                                     ....|....|....|....|....|....|....|....|....|....|....|....|....|....|....|....|....|....|....|....|

T. trichiura H. sapiens AM9929       ----------------------------------------------------------------------------------------------------
Trichuris sp. P. ursinus DGI G       ----------------------------------------------------------------------------------------------------
Trichuris sp. P. ursinus DGII        ----------------------------------------------------------------------------------------------------
Trichuris sp. P. ursinus DGIII       ----------------------------------------------------------------------------------------------------

T. trichiura H. sapiens GQ3015       ----------------------------------------------------------------------------------------------------
Trichuris sp. P. ursinus CP-GO       ----------------------------------------------------------------------------------------------------

T. trichiura N. gabriellae FM9       TGACCGCCGGTACCTGTTCCGTCCGGGCCTCAGCGGCTGCGGCAGCGTGGATCAGGCTGCCGTTAGCGCCGCCGGTTGCAGCCGACCGTGCCGTCGCGAG
T. trichiura C. guereza FM9919       TGACCGCCGGTACCTGTTCCGTCCGGGCCTCAGCGGCTGCGGCAGCGTGGATCAGGCTGCCGTTAGCGCCGCCGGTTGCAGCCGACCGTGCCGTCGCGAG

T. suis S. scrofa AM993016 Chi       ----------------------------------------------------------------------------------------------------
T. suis S. scrofa AM993012 Chi       ----------------------------------------------------------------------------------------------------

T. ovis O. aries JF680987 Irel       CTGA------------------------------------------------------------------------------------------------
T. discolor cattle AB367795 Ja       CTGA------------------------------------------------------------------------------------------------
T. discolor C. crispus AB36779       CTGA------------------------------------------------------------------------------------------------

                                             1010      1020      1030      1040      1050      1060      1070      1080      1090
                                     ....|....|....|....|....|....|....|....|....|....|....|....|....|....|....|....|....|....|....|....|
T. trichiura H. sapiens AM9929       --------------------------------------------------------------GCGCTAGTAGCATTCGAACTGCTGCTGTACGCGCAG--
Trichuris sp. P. ursinus DGI G       --------------------------------------------------------------GCGCTAGTAGCATTCGAACTGCTGCTGTACGCGCAG--
Trichuris sp. P. ursinus DGII        --------------------------------------------------------------GCGCTAGTAGCATTCGAACTGCTGCTGTACGCGCAG--
Trichuris sp. P. ursinus DGIII       --------------------------------------------------------------GCGCTAGTAGCATTCGAACTGCTGCTGTACGCGCAG--

T. trichiura H. sapiens GQ3015       ----------------------------------------------------------------------------------------------------
Trichuris sp. P. ursinus CP-GO       ----------------------------------------------------------------------------------------------------

T. trichiura N. gabriellae FM9       CTGTCATCACGTCGCCGACCGCTCCCGGTGGCGTTGATGCTGCGTCGTGCTATGTCGGTGAGGTGTAAAGAACGTCCGTTTGTCTCTTGAGCGCACCGAG
T. trichiura C. guereza FM9919       CTGTCATCACGTCGCCGACCGCTCCCGGTGGCGTTGATGCTGCGTCGTGCTATGTCGGTGAGGTGTAAAGAACGTCCGTTCGTCTCTTGAGCGCACCGAG

T. suis S. scrofa AM993016 Chi       ----------------------------------------------------------------------------------------------------
T. suis S. scrofa AM993012 Chi       ----------------------------------------------------------------------------------------------------

T. ovis O. aries JF680987 Irel       ----------------------------------------------------------------------------------------------------
T. discolor cattle AB367795 Ja       ----------------------------------------------------------------------------------------------------
T. discolor C. crispus AB36779       ----------------------------------------------------------------------------------------------------
```

```
                                          1110      1120      1130      1140      1150      1160      1170      1180      1190
                                 ....|....|....|....|....|....|....|....|....|....|....|....|....|....|....|....|....|....|....|....|

T. trichiura H. sapiens AM9929   -------------------------------------------------------------------------------------------GAAAGTACA
Trichuris sp. P. ursinus DGI G   -------------------------------------------------------------------------------------------GAAAGTACA
Trichuris sp. P. ursinus DGII    -------------------------------------------------------------------------------------------GAAAGTACA
Trichuris sp. P. ursinus DGIII   -------------------------------------------------------------------------------------------GAAAGTACA

T. trichiura H. sapiens GQ3015   -------------------------------------------------------------------------------------------ATGATGACA
Trichuris sp. P. ursinus CP-GO   -------------------------------------------------------------------------------------------ACGATGACA

T. trichiura N. gabriellae FM9   GAGAGACGGCTCGTCGCCGGTTGGATCTTCGTTAACGACCAATGCAGATAAGCTGCGCCTTCGTCGAACTGCGAAAACGGACTATTGACTGACGATGACA
T. trichiura C. guereza FM9919   GAGAGACGGCTCGTCGCCGGTTGGATCTTCGTTAACGACCAATGCAGATAAGCTGCGCCTTCGTCGAACTGCGAAAACGGACTACTGACTGACGATGACA

T. suis S. scrofa AM993016 Chi   -------------------------------------------------------------------------------------------ATGATGACA
T. suis S. scrofa AM993012 Chi   -------------------------------------------------------------------------------------------ATGATGACA

T. ovis O. aries JF680987 Irel   -------------------------------------------------------------------------------------------ACAGAGACA
T. discolor cattle AB367795 Ja   -------------------------------------------------------------------------------------------ATAGAGACA
T. discolor C. crispus AB36779   -------------------------------------------------------------------------------------------ATAGAGACA

                                          1210      1220      1230      1240      1250      1260      1270      1280      1290
                                 ....|....|....|....|....|....|....|....|....|....|....|....|....|....|....|....|....|....|....|....|

T. trichiura H. sapiens AM9929   TTCCGAACGGTGGATCACTTGGCTCGTAGATCGTTGAAGAACGACGTGACACTCGAGAATTGACGTGAATTGCAGACACACCGAACTTGAATACTTTGAA
Trichuris sp. P. ursinus DGI G   TTCCGAACGGTGGATCACTTGGCTCGTAGATCGTTGAAGAACGACGTGACACTCGAGAATTGACGTGAATTGCAGACACACCGAACTTGAATACTTTGAA
Trichuris sp. P. ursinus DGII    TTCCGAACGGTGGATCACTTGGCTCGTAGATCGTTGAAGAACGACGTGACACTCGAGAATTGACGTGAATTGCAGACACACCGAACTTGAATACTTTGAA
Trichuris sp. P. ursinus DGIII   TTCCGAACGGTGGATCACTTGGCTCGTAGATCGTTGAAGAACGACGTGACACTCGAGAATTGACGTGAATTGCAGACACACCGAACTTGAATACTTTGAA

T. trichiura H. sapiens GQ3015   TTCCGAACGGCGGATCACTTGGCTCGTAGGTCGTTGAAGAACGACGTGACACTCGAGAATTGATGTGAATTGCAGACACACTGAACTTGAATACTTTGAA
Trichuris sp. P. ursinus CP-GO   TTCCGAACGGCGGATCACTTGGCTCGTAGGTCGTTGAAGAACGACGTGACACTCGAGAATTGATGTGAATTGCAGACACACTGAACTTGAATACTTTGAA

T. trichiura N. gabriellae FM9   TTCCGAACGGCGGATCACTTGGCTCGTAGGTCGTTGAAGGACGACGTGACACTCGAGAATTGATGTGGATTGCAGACACACTGAACTTGAATACTTTGAA
T. trichiura C. guereza FM9919   TTCCGAACGGCGGATCACTTGGCTCGTAGGTCGTTGAAGGACGACGTGACACTCGAGAATTGATGTGAATTGCAGACACACTGAACTTGAATACTTTGAA

T. suis S. scrofa AM993016 Chi   TTCCGAACGGCGGATCACTTGGCTCGTAGATCGTTGAAGAACGACGTGACACTCGAGAATTGATGTGAATTGCAGACACACTGAACTTGAATACTTTGAA
T. suis S. scrofa AM993012 Chi   TTCCGAACGGCGGATCACTTGGCTCGTAGATCGTTGAAGAACGACGTGACACTCGAGAATTGATGTGAATTGCAGACACACTGAACTTGAATACTTTGAA

T. ovis O. aries JF680987 Irel   TTCCGAACGGCGGATCACTTGGCTCGTAGGTCGTTGAAGAACGACGTGACACTCGAGAATTGATGTGAATTGCAGACACACTGAACTTGAATACTTTGAA
T. discolor cattle AB367795 Ja   TTCCGAACGGCGGATCACTTGGCTCGTAGGTCGTTGAAGAACGACGTGACACTCGAGAATTGATGTGAATTGCAGACACACTGAACTTGAATACTTTGAA
T. discolor C. crispus AB36779   TTCCGAACGGCGGATCACTTGGCTCGTAGGTCGTTGAAGAACGACGTGACACTCGAGAATTGATGTGAATTGCAGACACACTGAACTTGAATACTTTGAA

                                                              5.8S
```

```
                                               1310      1320      1330      1340      1350      1360      1370      1380      1390
                                          ....|....|....|....|....|....|....|....|....|....|....|....|....|....|....|....|....|....|....|....|

T. trichiura H. sapiens AM9929            CGCACATTGCAGCGTCGATCAAAGATGTCGACGCTACGCCTGTCTGAGGGTCGTTAAGCATAATAGCGAATGCGCCGCTCAG------GCTACAG---GT
Trichuris sp. P. ursinus DGI G            CGCACATTGCAGCGTCGATCAAAGATGTCGACGCTACGCCTGTCTGAGGGTCGTTAAGCATAATAGCGAATGCGCCGCTCAG------GCTACAG---GT
Trichuris sp. P. ursinus DGII             CGCACATTGCAGCGTCGATCAAAGATGTCGACGCTACGCCTGTCTGAGGGTCGTTAAGCATAATAGCGAATGCGCCGCTCAG------GCTACAG---GT
Trichuris sp. P. ursinus DGIII            CGCACATTGCAGCGTCGATCAAAGATGTCGACGCTACGCCTGTCTGAGGGTCGTTAAGCATAATAGCGAATGCGCCGCTCAG------GCTACAG---GT

T. trichiura H. sapiens GQ3015            CGCACATTGCAGCGTCGACGAACCATGTCGACGCTACGCCTGTCTGAGGGTCGTTACGAAATAAAGCAAATGCGCCGCTCAGCAGGCTGCTACTGCCGCT
Trichuris sp. P. ursinus CP-GO            CGCACATTGCAGCGTCGACGAACCATGTCGACGCTACGCCTGTCTGAGGGTCGTTACGAAATAAAGCAAATGCGCCGCTCAGGCTGCTGCTGCTGCCTCT

T. trichiura N. gabriellae FM9            CGCACATTGCAGCGTCGACGAACCATGTCGACGCTACGCCTGTCTGAGGGTCGTTACGATGTAAAGCAAATGCGCCGCTCAG------GCTTCCGCCCGT
T. trichiura C. guereza FM9919            CGCACATTGCGGCGTCGACGAACCATGTCGACGCTACGCCTGTCTGAGGGTCGTTACGATGTAAAGCAAATGCGCCGCTCAG------GCTTCCGCCCGT

T. suis S. scrofa AM993016 Chi            CGCACATTGCAGCGTCGACGAAACATGTCGACGCTACGCCTGTCTGAGGGTCGTTACGAAATAAAGCAAATGCGCCGCTCAG------GCTTCGGCG-CT
T. suis S. scrofa AM993012 Chi            CGCACATTGCAGCGTCGACGAAACATGTCGACGCTACGCCTGTCTGAGGGTCGTTACGAAATAAAGCAAATGCGCCGCTCAG------GCTTCGGCG-CT

T. ovis O. aries JF680987 Irel            CGCACATTGCAGCGTCGATCAAACATGTCGGCGCTACGCCTGTCTGAGGGTCGTTATCGAC--ACGCAATTG---CGCTCAGGCTGACGCCACCGTGGCT
T. discolor cattle AB367795 Ja            CGCACATTGCAGCGTCGATCAAACATGTCGGCGCTACGCCTGTCTGAGGGTCGTTACCGAC--ACGCAATTG---CGCTCAGGCTGACGCCACCGTGGCT
T. discolor C. crispus AB36779            CGCACATTGCAGCGTCGATCAAACATGTCGGCGCTACGCCTGTCTGAGGGTCGTTACCGAC--ACGCAATTG---CGCTCAGGCTGACGCCACCGTGGCT

                                               1410      1420      1430      1440      1450      1460      1470      1480      1490
                                          ....|....|....|....|....|....|....|....|....|....|....|....|....|....|....|....|....|....|....|

T. trichiura H. sapiens AM9929            TGAG--------------------GTTGGTGGCGAGCA--CCGGACAAACCTGCATCCGCGC--G--CGAGC----GAGCGTGACGCCGCAGCTCCGTTG
Trichuris sp. P. ursinus DGI G            TGAG--------------------GTTGGTGGCGAGCA--CCGGACAAACCTGCATCCGCGC--G--CGAGC----GAGCGTGACGCCGCAGCTCCGTTG
Trichuris sp. P. ursinus DGII             TGAG--------------------GTTGGTGGCGAGCA--CCGGACAAACCTGCATCCGCGC--G--CGAGC----GAGCGTGACGCCGCAGCTCCGTTG
Trichuris sp. P. ursinus DGIII            TGAG--------------------GTTGGTGGCGAGCA--CCGGACAAACCTGCATCCGCGC--G--CGAGC----GAGCGTGACGCCGCAGCTCCGTTG

T. trichiura H. sapiens GQ3015            GGGA-----------------ACTAGCGGTAGCAAGCAGCGCGGGTACGGCTGCCCGTTGGTTGGTCTCAGC----GAGCGCGACGCCGCAGCTGCTCCT
Trichuris sp. P. ursinus CP-GO            TGGA-----------------GCTAGCGGTAGCAAGCAGCGCGGGTACGGTTGCCCGTTGGGCGC--TGAGC----GAGCGCGACGCCGCAGC------T

T. trichiura N. gabriellae FM9            TGGACATGCACTGTCCGTGCCGCTAGCGGTAGCAAGCAGCGCGGACGCGGCTACTCCCGTGT--C--TGAGC----GAGCGCGACGCCGCAGGTCGTCCT
T. trichiura C. guereza FM9919            TGGACATGCACTGTCCGTGCCGCTAGCGGTAGCAAGCAGCGCGGACGCGGCTACTCCCGTGT--C--TGAGC----GAGCGCGACGCCGCAGGTCGTCCT

T. suis S. scrofa AM993016 Chi            TGGACTTGCACGGTCCGTGCAGCTAGCTGTAGCAAGCGGCGCGGATACGGCTACCCGAGGCT--G--TGAGC----GAGCGCGACGCCGAGGCTCCTCCT
T. suis S. scrofa AM993012 Chi            TGGACTTGCACGGTCCGTGCAGCTAGCGGTAGCAAGCGGCGCGGATACGGCTACCCGAGGCT--G--TGAGC----GAGCGCGACGCCGAGGCTCCTCCT

T. ovis O. aries JF680987 Irel            TAGG-----------------------TACTTGAAGCTACGCG----TGGCGGCACGC---------TACGCCTCTAAGCGCTACGCTGC---------T
T. discolor cattle AB367795 Ja            TCGG-----------------------TACAGGAAGCTGCGCG----TGGCGGCACGA---------TGCGCCTCTGAGCGTTACGCTGC---------T
T. discolor C. crispus AB36779            TCGG-----------------------TACAGGAAGCTGCGCG----TGGCGGCACGA---------TGCGCCTCTGAGCGTTACGCTGC---------T
```

```
                                           1510      1520      1530      1540      1550      1560      1570      1580      1590
                                   ....|....|....|....|....|....|....|....|....|....|....|....|....|....|....|....|....|....|....|....|

T. trichiura H. sapiens AM9929     CCAG---CGAGCCG-CGATGGCA--------------------ACTGGTAGGCGG----AG---CAGCGGAGAGCGGCCAAGTCAGCGTAGGGCGAAGACT
Trichuris sp. P. ursinus DGI G     CCAG---CGAGCCG-CGATGGCA--------------------ACTGGTAGGCGG----AG---CAGCGGAGAGCGGCCAAGTCAGCGTAGGGCGAAGACT
Trichuris sp. P. ursinus DGII      CCAG---CGAGCCG-CGATGGCA--------------------ACTGGTAGGCGG----AG---CAGCGGAGAGCGGCCAAGTCAGCGTAGGGCGAAGACT
Trichuris sp. P. ursinus DGIII     CCAG---CGAGCCG-CGATGGCA--------------------ACTGGTAGGCGG----AG---CAGCGGAGAGCGGCCAAGTCAGCGTAGGGCGAAGACT

T. trichiura H. sapiens GQ3015     GCTGCTACTGGCAG-CGACGGCAG--GTGGCCGTCATCGCTGACAGGCAGCCGC----AGCTTCTGCGGAGAGCGGCTAACTCAGCGCAGTACGGAAGCT
Trichuris sp. P. ursinus CP-GO     GCTG---CTGGCAG-CGACGGCAG--GTGGCCGTCATCGCTGACAGGCAGCCGG----AG---CTGCGGAGAGCGGCCAACTCAGCGCAGTACGGAAGCT

T. trichiura N. gabriellae FM9     GCTG------GCAG-CGACGGCAG--GTGTTCGCTATCGCTGCCAGGCAGACGG----AC---CAGCGGAGAGCGGCTAACTCAGCGCAGCACGGAAGCT
T. trichiura C. guereza FM9919     GCTG------GCAG-CGACGGCAG--GTGTTCGCTATCGCTGCCAGGCAGACGG----AC---CAGCGGAGAGCGGCTAACTCAGCGCAGCACGGAAGCT

T. suis S. scrofa AM993016 Chi     GCTG---CTAGCAG-CGACGGCAG--GTGCCCGTCATCGCTGGCAGGCAGCCGG----AG---CTGCGGAGAGCGGCTAACTCAGCGCAGTACGGAAGCT
T. suis S. scrofa AM993012 Chi     GCTG---CTAGCAG-CGACGGCAG--GTGCCCGTCATCGCTGGCAGGCAGCCGG----AG---CTGCGGAGAGCGGCTAACTCAGCGCAGTACGGAAGCT

T. ovis O. aries JF680987 Irel     GCTG---ATGGCGGTCGAGTGCCGCTGGGTCAGCCGCAGCTGACCGGCAGCCGCTACTAG---CAGCGGAGAGCAGTCGGCTCGGCAGAGAACGTAGGCT
T. discolor cattle AB367795 Ja     GCTG---ATGGCGGTCGAGTGCCGCTGGGCCAGCCGCAGCTGGCCGGCAACCGCTACTAG---CAGCAGAGAGCAGTCGGCTCGGCGGAGAACGTAGGCC
T. discolor C. crispus AB36779     GCTG---ATGGCGGTCGAGTGCCGCTGGGCCAGCCGCAGCTGGCCGGCAACCGCTACTAG---CAGCAGAGAGCAGTCGGCTCGGCGGAGAACGTAGGCC

                                           1610      1620      1630      1640      1650      1660      1670      1680      1690
                                   ....|....|....|....|....|....|....|....|....|....|....|....|....|....|....|....|....|....|....|

T. trichiura H. sapiens AM9929     ACCCGACTTGGCTAC-CGGCCGCGCCGTCG---GCGTACAGC--------------AGTTGAGCAGGGAGCGGTGAC----CGCACGCGCT----CGTAG
Trichuris sp. P. ursinus DGI G     ACCCGACTTGGCTAC-CGGCCGCGCCGTCG---GCGTACAGC--------------AGTTGAGCAGGGAGCGGTGAC----CGCACGCGCT----CGTAG
Trichuris sp. P. ursinus DGII      ACCCGACTTGGCTAC-CGGCCGCGCCGTCG---GCGTACAGC--------------AGTTGAGCAGGGAGCGGTGAC----CGCACGCGCT----CGTAG
Trichuris sp. P. ursinus DGIII     ACCCGACTTGGCTAC-CGGCCGCGCCGTCG---GCGTACAGC--------------AGTTGAGCAGGGAGCGGTGAC----CGCACGCGCT----CGTAG

T. trichiura H. sapiens GQ3015     GCCCGAGTTGGCTATGTCGCTACATCGTCA---GCGTAAAGCCGGCGAACG---ACCGTTGACCACCGAGCGACCAC--TGCGGGCGCGAG----CGCAG
Trichuris sp. P. ursinus CP-GO     GCCCGAGTTGGCTATGTCGCTACATCGTCA---GCGTAAAGCGCGCGACCG---ACCGTTGACCACCGAGCAACCAT--CGCGTGCGCGAG----CGCAG

T. trichiura N. gabriellae FM9     GCCCGAGTCGGCTACGTCGCCGCGTCGTCG----------GC--GCGACCG---TCCGTTGACCACCGAGCGACCAT----CGCAATCGAG----CGCAG
T. trichiura C. guereza FM9919     GCCCGAGTCGGCTACGTCGCCGCGTCGTCG----------GC--GCGACCG---TCCGTTGACCACCGAGCGACCAT----CGCAATCGAG----CGCAG

T. suis S. scrofa AM993016 Chi     GCCCGAGTTGGCTACGTCGCTACATCGTCGTCAGCGTACAGC--GCGACTGAGTTGTTTTCACCACCGAGCGACCACCCCGCGCGCGCGAC----CGTAG
T. suis S. scrofa AM993012 Chi     GCCCGAGTTGGCTACGTCGCTACATCGTCGTCAGCGTACAGC--GCGACTGAGTTGTTTTCACCACCGAGCGACCACCCCGCGCGCGCGCGCGACCGTAG

T. ovis O. aries JF680987 Irel     TTCCGAGTCGGCTTTTTACCCTCCTAATCG--------CGGC------------GCAGGCGACCATCGAGAAACGGC------------------TGCAA
T. discolor cattle AB367795 Ja     TTCCGAGTCGACTTTTCATGCTCGTAATCG--------CGGC------------GCAGGCGACCATCGAGAAACGGC------------------TGCAA
T. discolor C. crispus AB36779     TTCCGAGTCGACTTTTCATGCTCGTAATCG--------CGGC------------GCAGGCGACCATCGAGAAACGGC------------------TGCAA
```

```
                                      1710      1720      1730      1740      1750      1760      1770      1780      1790
                                ....|....|....|....|....|....|....|....|....|....|....|....|....|....|....|....|....|....|....|....|

T. trichiura H. sapiens AM9929  CAGCAAGTGTTCGTCGTC-GTTGCACGG---CAGCAGCAGCAGCAGCAGCAGTCGAC--GACGACGACGACGAAACCGTTCGCCTTGCTGCG------GC
Trichuris sp. P. ursinus DGI G  CAGCAAGTGTTCGTCGTA-GTTGCACGGCAGCAGCAGCAGCGGCAGCAGCAGTCGAC--GACGACGACGACGAAACCGTTCGCCTTGCTGCG------GC
Trichuris sp. P. ursinus DGII   CAGCAAGTGTTCGTCGTA-GTTGCACGGCAGCAGCAGCAGCGGCAGCAGCAGTCGAC--GACGACGACGACGAAACCGTTCGCCTTGCTGCG------GC
Trichuris sp. P. ursinus DGIII  CAGCAAGTGTTCGTCGTA-GTTGCACGGCAGCAGCAGCAGCGGCAGCAGCAGTCGAC-----GACGACGACGAAACCGTTCGCCTTGCTGCG------GC
                                                 SNP-6            DG-VNTR3                   DG-VNTR4

T. trichiura H. sapiens GQ3015  TCGTCCGTCTTCGTCGCC-GCCCCCTAG-------ATCGACGGCAGCGGCAGTCGACTAGAGGACGACGACGGCGTCT---GC--TGCTACGCGCTCTGC
Trichuris sp. P. ursinus CP-GO  TCGTCCGTCTTCGTCGCC-GCCCCCTAG-------ATCGACGGCAGCGGCAGTCGACTAGAAGACGACGACGGCGTCT---GC--TGCTACGCGCTCTGC

T. trichiura N. gabriellae FM9   TCGTCCGTCCTCGTTGCC-GCTCCCTAG-------ATCGACGGCAGTGGCAGTCGACTAGTAGACGACGACGACGGCTGCTGC--TGCTACGCGATCCGC
T. trichiura C. guereza FM9919   TCGTCCGTCCTCGTTGCC-GCTCCCTAG-------ATCGACGGCAGTGGCAGTCGACTAGTAGACGACGACGACGGCTGCTGC--TGCTACGCGATCCGC

T. suis S. scrofa AM993016 Chi  TCGTCCGTCTTCGTCACC-GGCCCCTAG-------ATCGACGGCAGCAGCAGTCGACTAGTAGACGACGACG---------GC--TGCTACGCGCTCCGC
T. suis S. scrofa AM993012 Chi  TCGTCCGTCTTCGTCACCGGCCCCCTAG-------ATCGACGGCAGCAGCAGTCGACTAGTAGACGATGACG---------GC--TGCTACGCGCTCCGC

T. ovis O. aries JF680987 Irel  -----AGTCGTCGGCATC---------------------------GAAACAGTCGACTTTCAGTCG-CGGCAA--------GCGTTGAT-----------
T. discolor cattle AB367795 Ja  -----AGTCGTCGGCATC---------------------------GAAACAGTCGACTTTCAGTCG-CGGCAA--------GCGTTGAT-----------
T. discolor C. crispus AB36779  -----AGTCGTCGGCATC---------------------------GAAACAGTCGACTTTCAGTCG-CGGCAA--------GCGTTGAT-----------
                                      1810      1820      1830      1840      1850      1860      1870      1880      1890
                                ....|....|....|....|....|....|....|....|....|....|....|....|....|....|....|....|....|....|....|

T. trichiura H. sapiens AM9929  GGCGGGCACCGCTCGACCGACGACGGCGGCAAACGTTGCTGACACAC---TGCTACCGGTCAGGCGGCGAGCTGCCGGTA---GCGGCGTCTTTGCCGTT
Trichuris sp. P. ursinus DGI G  GGCGGGCACCGCTCGACCGACGACGGCGGCAAACGTTGCTGACACAC---TGCTACCGGTCAGGC---GAGCTGCCGGTA---GCGGCGTCTTTGCCGTT
Trichuris sp. P. ursinus DGII   GGCGGGCACCGCTCGACCGACGACGGCGGCAAACGTTGCTGACACAC---TGCTACCGGTCAGGC---GAGCTGCCGGTA---GCGGCGTCTTTGCCGTT
Trichuris sp. P. ursinus DGIII  GGCGGGCACCGCTCGACCGACGACGGCGGCAAACGTTGCTGACACAC---TGCTACCGGTCAGGC---GAGCTGCCGGTA---GCGGCGTCTTTGCCGTT
                                                                                              DG-VNTR5

T. trichiura H. sapiens GQ3015  AGCG------GCGATCTCGGCTGCGACGGCAAACGTTGCTGACACACCGCTGCTACCACTTGCTGCTGCTGCTGCTGGTAGCGGCGGCGTCTTTGCCGTA
Trichuris sp. P. ursinus CP-GO  GGCGGCGGCAGCGATCTCGGCTGCGACGGCAAACGTTGCTGACACACCGTTACTACCACT------TGTTGCTGCTGGTAGCAGCGGCGTCTTTGCCGTA
                                                                                              CP-GOB VNTR2

T. trichiura N. gabriellae FM9   GG---------------------CGGCGGCAAACGTTGCTGACACACCGCTACTACCGCT------TGCTGCTGCTGGTAGCGGCGGCGTCTTTGCCGTA
T. trichiura C. guereza FM9919   GG---------------------CGGCGGCAAACGTTGCTGACACACCGCTACTACCGCT------TGCTGCTGCTGGTAGCGGCGGCGTCTTTGCCGTA

T. suis S. scrofa AM993016 Chi  GGCG------GTCGTGGCTGCGCCGGCGGCAAACGTTGCTGACACTCCGCTACTACCACG---------TGCTGCTGGTAGCGGCGGCGTCTTTGCCGTA
T. suis S. scrofa AM993012 Chi  GGCG------GTCGTGGCTGCGCCGGCGGCAAACGTTGCTGACACTCCGCTACTACCACG---------TGCTGCTGGTAGCGGCGGCGTCTTTGCCGTA

T. ovis O. aries JF680987 Irel  ---------------------TAAGGCAGTACGC---GCGGGCGTCCTGTCCTTGCCGGCTGCCGGTTTGGCTGCTGCT--CGGTGGAAGCTAG---GTG
T. discolor cattle AB367795 Ja  ---------------------TGAGGCAGTACGC---GCAGGCGTCCTGTCCTTGCCGGCTGCCCGTTTGGCTGCTGCT--CGGTGGAAGCTAG---TTG
T. discolor C. crispus AB36779  ---------------------TGAGGCAGTACGC---GCAGGCGTCCTGTCCTTGCCGGCTGCCCGTTTGGCTGCTGCT--CGGTGGAAGCTAG---TTG
```

```
                                            1910      1920      1930      1940      1950      1960
                                    ....|....|....|....|....|....|....|....|....|....|....|....|...

T. trichiura H. sapiens AM9929      CGTCA---ACTAGCGGTTCGCCGCG---TGGCTC--CACCCGTAC----GTCTCCTCGATGTT
Trichuris sp. P. ursinus DGI G      CGTCA---ACTAGCGGTTCGCCGCG---TGGCTC--CACCCGTAC----GTCTCCTCGATGTT
Trichuris sp. P. ursinus DGII       CGTCA---ACTAGCGGTTCGCCGCG---TGGCTC--CACCCGTAC----GTCTCCTCGATGTT
Trichuris sp. P. ursinus DGIII      CGTCA---ACTAGCGGTTCGCCGCG---TGGCTC--CACCCGTAC----GTCTCCTCGATGTT

T. trichiura H. sapiens GQ3015      CGCTGGTCAGTCACGG-CCGCAACG---CCACTG--CGCTCCCGTTGTTGTCTCTTCG-TGTT
Trichuris sp. P. ursinus CP-GO      CGCTGGTCACCGTCGG-CCGCTACG---CCACTG--CGCTCTCGTTGTTGTGTCTTCG-TGTT

T. trichiura N. gabriellae FM9      CGCTGGTCACTCTCGG-CCGCCGCAAAGCGACTG--CGCTCCCGTTCTTGT-TGTTCG-TGTT
T. trichiura C. guereza FM9919      CGCTGGTCACTCTCGG-CCGCCGCAAAGCGACTG--CGCTCCCGTTCTTGT-TGTTCG-TGTT

T. suis S. scrofa AM993016 Chi      CAGTGGTGACTCTCGG-CCGCCGCAA--CAAGTG--CGCTCCCGTTGTTGT----TCG-TGTT
T. suis S. scrofa AM993012 Chi      CAGTGGTGACTCTCAG-CCGCCGCAA--CGAGTG--CGCTCCCGTTGTTGT----TCG-TGTT

T. ovis O. aries JF680987 Irel      CGCTGGT----------CCGCGGAA---CGAGTGGTCGCTTCTGC--------------TGTT
T. discolor cattle AB367795 Ja      CGCTGGT----------CCGCAGAA---CGAGTGGTCGCTTCTGC--------------TGTT
T. discolor C. crispus AB36779       CGCTGGT----------CCGCAGAA---CGAGTGGTCGCTTCTGC--------------TGTT
```
